# Functional analysis after rapid degradation of condensins and 3D-EM reveals chromatin volume is uncoupled from chromosome architecture in mitosis

**DOI:** 10.1242/jcs.210187

**Published:** 2018-02-15

**Authors:** Kumiko Samejima, Daniel G. Booth, Hiromi Ogawa, James R. Paulson, Linfeng Xie, Cara A. Watson, Melpomeni Platani, Masato T. Kanemaki, William C. Earnshaw

**Affiliations:** 1 Wellcome Centre for Cell Biology, University of Edinburgh, King's Buildings, Max Born Crescent, Edinburgh EH9 3BF, Scotland, UK; 2 Department of Chemistry, University of Wisconsin-Oshkosh, 800 Algoma Blvd, Oshkosh, WI 54901, USA; 3 Division of Molecular Cell Engineering, National Institute of Genetics, ROIS, and Department of Genetics, SOKENDAI, Yata 1111, Mishima, Shizuoka 411-8540, Japan

**Keywords:** CDK1, Chromosome, DT40, Mitosis, Auxin inducible degron system, Condensin

## Abstract

The requirement for condensin in chromosome formation in somatic cells remains unclear, as imperfectly condensed chromosomes do form in cells depleted of condensin by conventional methodologies. In order to dissect the roles of condensin at different stages of vertebrate mitosis, we have established a versatile cellular system that combines auxin-mediated rapid degradation with chemical genetics to obtain near-synchronous mitotic entry of chicken DT40 cells in the presence and absence of condensin. We analyzed the outcome by live- and fixed-cell microscopy methods, including serial block face scanning electron microscopy with digital reconstruction. Following rapid depletion of condensin, chromosomal defects were much more obvious than those seen after a slow depletion of condensin. The total mitotic chromatin volume was similar to that in control cells, but a single mass of mitotic chromosomes was clustered at one side of a bent mitotic spindle. Cultures arrest at prometaphase, eventually exiting mitosis without segregating chromosomes. Experiments where the auxin concentration was titrated showed that different condensin levels are required for anaphase chromosome segregation and formation of a normal chromosome architecture.

This article has an associated First Person interview with the first author of the paper.

## Abstract (precis)

**Summary:** Rapid condensin depletion reveals that different condensin levels are required for mitotic chromosome architecture and segregation. Condensin is not required for chromatin volume compaction during mitosis.

## INTRODUCTION

As cells enter mitosis, the chromatin volume is compacted by 2–3-fold as it undergoes a dramatic re-organization (Llères et al., 2009; Martin and Cardoso, 2010; Vagnarelli and Earnshaw, 2012). Rod-shaped mitotic chromosomes form and chromosome scaffold proteins are localized along the sister chromatid axes (Paulson and Laemmli, 1977; Lewis and Laemmli, 1982; Earnshaw et al., 1985; Gasser et al., 1986; Saitoh et al., 1994; Samejima et al., 2012). These proteins include topoisomerase IIα, chromokinesin KIF4 and structural maintenance of chromosomes 2 (SMC2), a core subunit of both condensin complexes (Earnshaw et al., 1985; Gasser and Laemmli, 1986; Saitoh et al., 1994; Hirano and Mitchison, 1994; Hirano et al., 1997; Ono et al., 2003; Samejima et al., 2012).

Condensin loss is reported to lead to anaphase chromosome mis-segregation with masses of lagging chromosomes blocking cytokinesis in yeasts, *Drosophila melanogaster*, nematodes and vertebrate cells (Saka et al., 1994; Steffensen et al., 2001; Hagstrom et al., 2002; Hudson et al., 2003). Mitotic chromosome structural defects are also commonly observed, but to varying degrees in different experimental systems (Hirano and Mitchison, 1994; Hirano et al., 1997; Saka et al., 1994; Bhat et al., 1996; Steffensen et al., 2001; Hagstrom et al., 2002; Somma et al., 2003; Hudson et al., 2003; Coelho et al., 2003; Vagnarelli et al., 2006; Maddox et al., 2006; Samoshkin et al., 2009; Houlard et al., 2015; Piskadlo et al., 2017). At one extreme, recognizable condensed chromosomes do not form in *Xenopus* egg extracts immuno-depleted of condensins (Hirano and Mitchison, 1994; Hirano et al., 1997), in *Schizosaccharomyces pombe* temperature-sensitive condensin mutants at the restrictive temperature (Saka et al., 1994; Sutani et al., 1999), in mouse meiosis I oocytes depleted of condensin II, and *Drosophila* embryos in which condensin I is acutely inactivated by TEV protease-mediated cleavage (Houlard et al., 2015; Piskadlo et al., 2017). These observations suggest that condensins are essential for assembly and maintenance of mitotic and meiotic chromosome structure.

At the other extreme, vertebrate cells depleted of condensins using conditional knockouts or siRNA exhibit relatively moderate defects in chromosome structure (Hudson et al., 2003; Ono et al., 2003, 2004; Vagnarelli et al., 2006; Samoshkin et al., 2009). Individual chromosomes are seen, but they are wider and appear to lack the structural rigidity seen in wild-type chromosomes. These inconsistent phenotypes among different experimental systems pose a ‘condensin paradox’ (Gassmann et al., 2004), suggesting that condensin might not be universally required for mitotic chromosome formation.

One possible explanation was that the effect of condensin depletion might vary in different experimental systems and other factors might contribute to shape mitotic chromosomes in vertebrate somatic cells (Vagnarelli et al., 2006; Samejima et al., 2012; Takagi et al., 2017). Alternatively, differences in the kinetics of condensin depletion and/or in the residual amount of condensin could correlate with the extent of defects in mitotic chromosome formation. The latter hypothesis is supported by observations showing that more severe chromosomal defects are associated with systems where condensins are either pre-depleted or acutely inactivated (Hirano and Mitchison, 1994; Saka et al., 1994; Hirano et al., 1997; Sutani et al., 1999; Houlard et al., 2015; Piskadlo et al., 2017), while milder chromosomal defects are reported when condensin is gradually lost by natural turnover over more than one cell cycle after synthesis of new protein was halted (Hudson et al., 2003; Ono et al., 2003, 2004; Vagnarelli et al., 2006; Samoshkin et al., 2009). The milder chromosomal defects might be explained by cellular adaptation to the gradual loss and/or incomplete depletion of condensin (see e.g. Wood et al., 2016). Furthermore, the various mitotic defects observed might even result from non-mitotic functions of condensin (Hirano, 2016). Taken together, these observations suggest that rapid and controllable depletion of condensin in vertebrate cells might more accurately reveal its true mitotic function(s) and differentiate between the above hypotheses.

Rapid protein depletion can be achieved using an auxin-inducible degron (AID) system (Nishimura et al., 2009; Kanemaki, 2013). The plant hormone auxin enhances the affinity of the plant-specific F-box protein *Oryza sativa* (Os)TIR1 for the AID tag *Arabidopsis thaliana* (At)IAA17. In the presence of auxin, tagged target proteins become poly-ubiquitylated and are degraded rapidly via the ubiquitin-proteasome pathway. It can take as little as 1 h to deplete a target protein in vertebrate cells. Furthermore, the AID system has allowed us to study cells partially depleted of condensin by titrating the amount of auxin (Nishimura et al., 2009). TEV protease cleavage of condensin is even more rapid, requiring only 15 min to fully cleave the target protein (Piskadlo et al., 2017). However, titration of target protein levels is difficult or impossible using TEV protease cleavage or Cre/loxP-mediated inactivation of the target gene (Houlard et al., 2015; Piskadlo et al., 2017). Furthermore, protein fragments produced by TEV protease cleavage could conceivably exert unexpected biological functions.

A fundamental difficulty with studying mitotic chromosome formation is that chromosome morphology changes on a minute-by-minute basis as cells enter mitosis. However, prophase cells comprise less than 1% of the population in asynchronous cultures. Thus, cell synchrony protocols are needed. Importantly, commonly used nocodazole or colcemid treatments are not suitable for studying chromosome morphology, as both treatments delay cells in prometaphase and induce hyper-condensation of the chromosomes (Hudson et al., 2003; Fernandes et al., 2013).

An alternative strategy is to synchronize cultures before they enter mitosis. Regulated CDK1 activity is essential for mitotic entry and progression. Manipulation of this activity can be used to enrich for mitotic cells (Nurse and Bissett, 1981; Vassilev et al., 2006; Smith et al., 2011). To this end, an analogue-sensitive cyclin-dependent kinase 1 (CDK1^as^) system was established to specifically inhibit CDK1 activity (Hochegger et al., 2007). CDK1^as^ has a mutation in the ATP-binding pocket allowing the binding of 1NMPP1, an ATP analog with a bulky side chain (Bishop et al., 2000). Addition of 1NMPP1 blocks CDK1^as^ cells in late G_2_. Upon washout of the drug, the cells swiftly enter mitosis.

Here, we induced a rapid SMC2 degradation in highly synchronized cultures and undertook an analysis with three-dimensional electron microscopy (3D-EM) to probe the function of condensin in mitotic chromosome structure. We find that mitotic chromatin is compacted to a volume similar to that of wild-type chromosomes, but that the chromosome morphology is highly aberrant in cells acutely depleted of condensin. We also find that differing levels of condensin are required for normal chromosome architecture, sister chromatid axis formation and chromosome segregation at anaphase.

## RESULTS

### The SMC2-AID cell line to study the mitotic roles of condensin

Defining the roles of condensin in vertebrate cells during mitosis has been difficult due to relatively mild defects observed following depletion of condensin subunits in mitotic cells of numerous species, compared to defects seen *in vitro*, in yeasts, in meiotic mouse oocytes and in fly embryos. This could be explained by there being variable levels of residual condensin in different experimental systems or by the cells adapting to the gradual loss of condensin. We hypothesized that rapid depletion of a key condensin subunit might resolve this issue. In order to test this hypothesis, we modified SMC2 (a subunit common to condensin I and II) to be rapidly depleted in chicken DT40 cells. Both condensin I and II have important roles in mitotic chromosome assembly in chicken DT40 cells similar to what is seen in human cultured cells (Ono et al., 2003; Green et al., 2012), and this allowed us to directly compare the defects with those of our chicken DT40 conditional SMC2 knockouts (SMC2^ON/OFF^; denoted as SMC2^OFF^ treated with Dox >30 h, or SMC2^ON^ with no Dox) (Hudson et al., 2003).

**Fig. 1.**
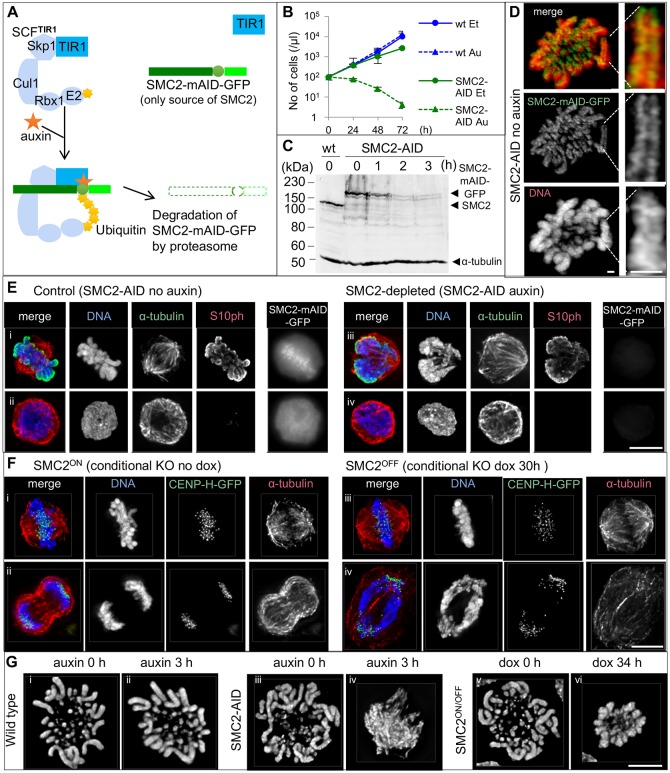
**Rapid depletion of SMC2–mAID–GFP upon auxin treatment results in a severely defective mitotic chromosome formation.** (A) Diagram introducing SMC2-AID cells. Auxin addition recruits the mAID tag to the SCF^TIR1^ complex, resulting in rapid degradation of SMC2–mAID–GFP via the proteasome pathway. (B) Growth curve of wild-type (wt) cells and SMC2-AID cells treated with either ethanol (Et, solvent) or 125 µM auxin (Au). Data are plotted as mean±s.d. (*n*=4). (C) Immunoblot analysis of asynchronous wild-type and SMC2-AID cells. SMC2-AID cells were treated with 125 µM auxin for 0–3 h. SMC2 and SMC2–mAID–GFP were detected with an anti-SMC2 antibody. α-tubulin was used as a loading control. (D) Live-cell imaging of a SMC2-AID cell with a Zeiss Airyscan microscope. DNA was stained with SiR-DNA. SMC2–mAID–GFP concentrated along the axis of sister chromatids. Scale bars: 1 µm. (E) SMC2-AID cells treated with ethanol (solvent) (i,ii) or auxin for 3 h (iii,iv) were fixed with 4% formaldehyde, and stained for α-tubulin (green), H3S10ph (red) and DNA (blue). The GFP signal was undetectable (iii,iv) and the shape of mitotic chromosomes (iii) was highly abnormal in SMC2-depleted cells. Scale bar: 5 µm. (F) SMC2^ON/OFF^/CENP-H–GFP cells (Vagnarelli et al., 2006) converted into CDK1^as^ were treated with doxycycline for 0 or 30 h, and stained in metaphase and anaphase for DNA (blue), CENP-H–GFP (green) and α-tubulin (red). (G) Wild-type/CDK1^as^ cells (0 or 3 h auxin treatment), SMC2-AID/CDK1^as^ cells (0 or 3 h auxin treatment) cells and SMC2^ON/OFF^/CDK1^as^ cells (0 or 34 h doxycycline treatment) were fixed with cold methanol/acetic acid and stained for DNA. More examples are shown in Fig. S1B. Scale bars: 5 µm.

Using an AID system, we established the SMC2-AID cell line expressing a SMC2 cDNA fused with a minimal AID degron and GFP tag as the sole source of SMC2 together with the auxin-responsive F-box protein TIR1 (Fig. 1A and see Materials and Methods). SMC2-AID cells proliferated well in the absence of auxin, but died when it was added to the medium (Fig. 1B). In contrast, addition of auxin did not affect the growth rate of wild-type cells. The mitotic index (MI), mitotic profile and cell cycle profile of the SMC2-AID cells were similar to those of wild-type cells without auxin (Figs S1A, S2C). SMC2–mAID–GFP (mAID denotes a minimal AID tag) was expressed at a level similar to the endogenous SMC2 of wild-type cells (Fig. 1C) and localized correctly along the axis of sister chromatids in living cells (Fig. 1D). Prometaphase chromosomes of SMC-AID cells appeared indistinguishable from those of wild-type cells in the absence of auxin (Fig. 1G; Fig. S1B).

### Chromosomes condense into a single mass following rapid SMC2 depletion

**Fig. 2.**
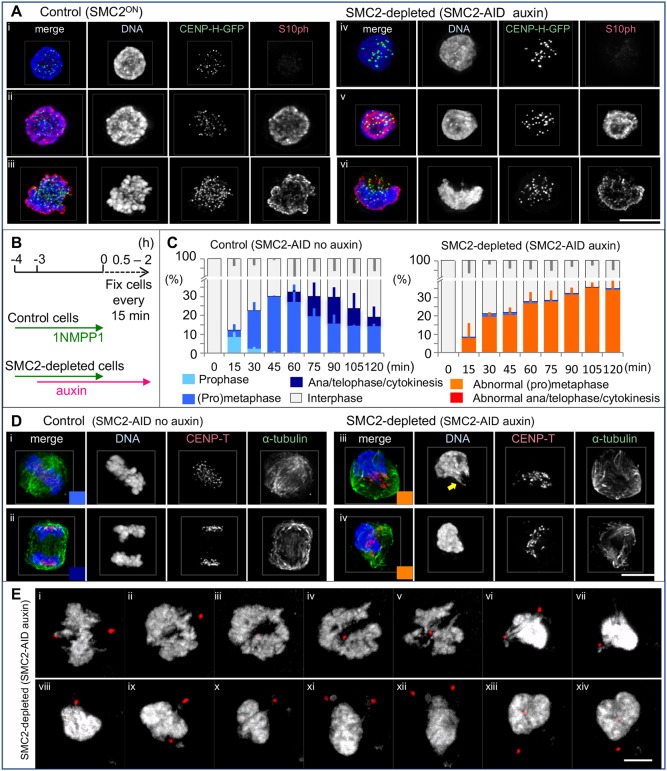
**SMC2-depleted cells accumulate at prometaphase with a single chromosome mass.** (A) H3S10ph (red) was not affected by SMC2 depletion. Interphase cells (i), prophase cells (ii) and prometaphase cells (iii). Control, SMC2^ON^/CDK1^as^/CENP-H-GFP cells. SMC2-depleted, SMC2-AID/CDK1^as^/CENP-H-GFP cells. (B) Experimental timeline for auxin treatment. After 1NMPP1 washout, SMC2-AID/CDK1^as^ cells were fixed with 4% formaldehyde and stained with the indicated antibodies. (C) Cell cycle and morphological analysis. >200 cells were counted for each time point for each treatment treated as in B. Data are plotted as mean±s.d. (*n*=3). (D) Representative images of control cells and SMC2-depleted cells stained with anti-CENP-T and -α-tubulin antibodies at different stages of mitosis. The colored box at the bottom right corners of merge panels correspond to the stages of mitosis shown in C. SMC2-depleted mitotic cells have a single chromosome mass adjacent to a malformed mitotic spindle. A stretched chromatin fiber is highlighted by the arrow in iii, right. Normal prometaphase-like chromosomes were occasionally observed within the auxin-treated cell population but all of those cells were SMC2–mAID–GFP positive. (E) Stills from live-cell imaging of SMC2-AID/CDK1^as^ cells expressing PACT–RFP (red) treated with auxin for 3–6 h. DNA was stained with SiR-DNA. 3D image stacks were collected at 0.4-µm *z* increments on a Zeiss Airyscan microscope every 5 min. SMC2-AID/CDK1^as^ cells exited mitosis without apparent chromosome segregation. Chromosomes appeared to be decondensed with centrosomes positioned close by in later time points (xii–xiv). The control (no auxin) is shown in Fig. S2D. Scale bars: 5 µm.

Auxin addition induced the rapid depletion of SMC2–mAID–GFP in asynchronous cell cultures. After a 3 h auxin treatment, the amount of SMC2–mAID–GFP protein fell to ∼5% of the wild-type level, the number of GFP-positive cells declined, and the GFP signal was lost (Fig. 1C,E; Fig. S1C). Chromatin of mitotic cells depleted of SMC2 clustered in a compact mass in which single chromosomes could not be resolved (Fig. 1E–G; Fig. S1B). The chromosome morphological defects seen in SMC2-AID cells treated with auxin were much more severe than those in SMC2^OFF^ (a conventional conditional knockout) cells treated with doxycycline for 30–34 h. SMC2^OFF^ chromosomes were wider and fuzzier than controls, but individual chromosomes were still visible (Figs 1G and 2Ai; Fig. S1B). Furthermore, SMC2^OFF^ cells underwent aberrant anaphase with chromosome bridges as previously reported (Fig. 1Fiv). In previous reports, chromosome mis-segregation at anaphase was the most prominent phenotypic defect after condensin depletion or inactivation (Saka et al., 1994; Steffensen et al., 2001; Hagstrom et al., 2002; Hudson et al., 2003). We were therefore surprised to observe no anaphase or telophase cells following rapid SMC2 depletion (Fig. 2C).

The strong chromosome phenotype observed after rapid condensin depletion made it difficult to determine the mitotic stage at which the cells arrested. We therefore stained a sub-clone of SMC2-AID cells, expressing CENP-H–GFP from the endogenous allele, with antibodies to histone H3S10ph (histone H3 phosphorylated on S10) and INCENP following treatment with vehicle (ethanol) or auxin for 3 h. The signal intensity of H3S10ph is normally high on prometaphase and metaphase chromosomes and progressively decreases after anaphase onset. INCENP is a scaffolding and regulatory subunit of the chromosome passenger complex that localizes to chromosome arms and centromeres in early mitosis, then transfers to the central spindle and cleavage furrow during mitotic exit (Carmena et al., 2012).

The H3S10ph level on the SMC2-depleted chromosome mass resembled that on control prometaphase or metaphase chromosomes (Figs 1Ei,iii, 2Aii,iii). Furthermore, INCENP localized on the SMC2-depleted chromosome mass and not on the spindle (Fig. S2A). These observations suggest that, after auxin treatment, SMC2-depleted cells with chromosome clusters are in early mitosis.

These results in SMC2-AID cells treated with auxin revealed that condensin is required to shape and resolve individual mitotic chromosomes in cultured vertebrate cells. In addition, phenotypic differences between SMC2^OFF^ cells and SMC2-AID cells depleted of SMC2 supported our hypothesis that the rate of condensin loss influences the final chromosome morphology in somatic cells.

### Use of chemical genetics to obtain mitotically synchronized cells lacking condensin

In order to obtain DT40 cultures progressing through mitosis in a synchronous wave, we introduced a CDK1^as^ cDNA into SMC2-AID cells and then disrupted the endogenous CDK1 alleles by using CRISPR/Cas9 technology. 1NMPP1, a bulky ATP analogue, inhibits the mutant CDK1^as^, but no other kinases, in these cells. As expected, 1NMPP1 did not prevent cells from progressing through G_1_, S and G_2_ phases; however, it prevented G_2_ cells from entering mitosis, and forced pre-existing mitotic cells to exit mitosis prematurely (Hochegger et al., 2007). No mitotic cells were detected 30 min after addition of 1NMPP1 (Fig. S2B). Subsequent washout of 1NMPP1 from the arrested G_2_ cells triggered a rapid and near synchronous wave of mitotic entry and exit (Fig. 2C; Fig. S2C).

Introduction of the CDK1^as^ circuit into SMC2-AID cells (termed SMC2-AID/CDK1^as^ cells) allowed us to investigate the effects of depleting condensin either just prior to mitosis or during a mitotic arrest while avoiding confounding effects from previous cell divisions.

A typical experimental time line using SMC2-AID/CDK1^as^ cells is illustrated in Fig. 2B (see Materials and Methods). All control cells (no auxin) were in interphase at *t*=0 min. The mitotic index of the control cells increased gradually from *t*=15 min, peaked at *t*=60 min, then decreased thereafter (Fig. 2C). Prophase cells were observed at *t*=15 min but were mostly gone by 30 min. Most mitotic cells at *t*=30–60 min were in prometaphase or metaphase (Fig. 2C). Cells in anaphase and telophase were seen at *t*=60–120 min. Furthermore, G_1_ cells were detected 1 h after release from a 1NMPP1 block by DNA content analysis (Fig. S2C). Thus, mitosis lasts ∼1 h in control cells after release from a 4 h 1NMPP1 block.

### SMC2-depleted cells accumulate at an abnormal prometaphase then exit mitosis without segregating chromosomes

Control cells (SMC2-AID/CDK1^as^ with no auxin) exhibited prophase chromosome condensation within 15 min of the 1NMPP1 washout (Fig. 2C). In contrast, SMC2-depleted cells did not show chromatin condensation until after nuclear envelope breakdown, as previously reported with RNAi or conditional knockouts of condensin (Hudson et al., 2003; Hirota et al., 2004; Ono et al., 2004).

The lack of prophase condensation could explain the slightly lower mitotic index of SMC2-depleted cells at *t*=15 min (Fig. 2C). However, the mitotic index caught up with that of control cells by *t*=30–60 min and continued to increase throughout the time course. No recognizable anaphase or telophase was observed in SMC2-depleted cells over the 2 h time course of this experiment confirming the results in asynchronous culture (Fig. 2C,D). Nevertheless, huge polyploid interphase and mitotic cells were seen following 2 days of auxin treatment (Fig. S2E), suggesting that these cells somehow exited the previous mitosis and continued to cycle.

Live-cell imaging showed that SMC2-depleted cells exited mitosis without properly segregating chromosomes (Fig. 2E) although control cells did so correctly (Fig. S2D). This explains why we did not observe anaphase or telophase cells following rapid SMC2 depletion.

### SMC2-depleted cells have a malformed mitotic spindle with the chromosome mass to one side

Control chromosomes congress to the middle of a bipolar spindle during late prometaphase and metaphase (Figs 1E, 2Di, 3A,B; Movie 1). In contrast, the single amorphous mass formed by SMC2-depleted chromosomes was largely excluded from the spindle. A few chromatin fibers protruded from this mass and extended into the spindle with centromeres at their tips (Figs 2Di, 3Aii; Movie 2). This suggests that chromosome arms did not follow as their centromeres congressed to the spindle midzone.

**Fig. 3.**
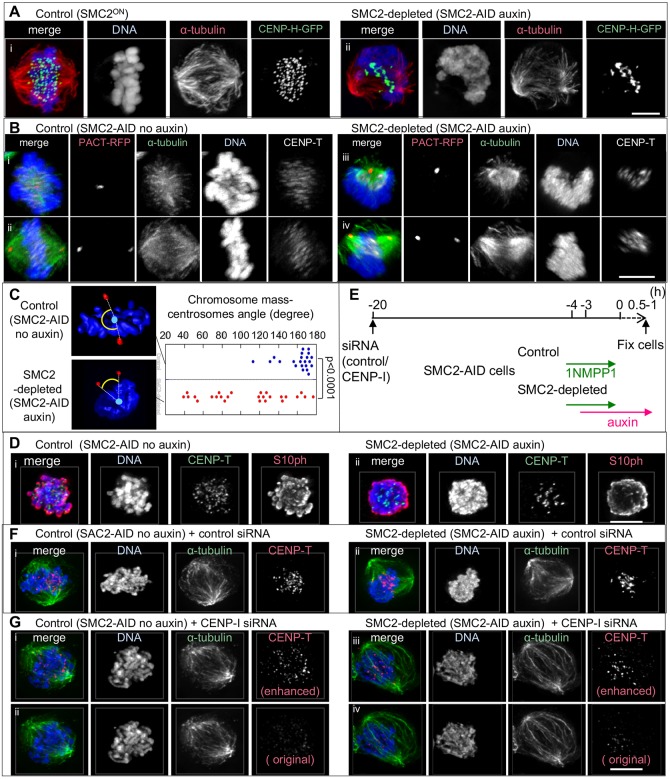
**Malformed and mis-positioned chromosomes and mitotic spindle in SMC2-depleted cells.** (A) SMC2^ON^/CDK1^as^/CENP-H-GFP cells and SMC2-AID/CDK1^as^/CENP-H-GFP (treated with auxin) were fixed with formaldehyde and stained for α-tubulin and DNA. Images were acquired by structured illumination microscopy. (B) SMC2-AID/CDK1^as^ cells expressing PACT-RFP were treated with 1NMPP1 for 4 h with or without auxin for 3 h. These cells were fixed with formaldehyde 1 h after 1NMPP1 washout and stained for α-tubulin (green), CENP-T (white), DNA (blue). Cells were 3D-rotated so that two PACT-RFP signals overlapped (i,iii) or were positioned in parallel (ii,iv). Note: Signals appeared to be fuzzier and stretched due to 3D-rotation. (C) SMC2-AID/CDK1^as^ cells expressing PACT-RFP were treated as (B) and stained for DNA. Angles between chromosomes and centrosomes were calculated as described in Materials and Methods. Control (22 cells), SMC2-depleted (26 cells). *P* value was <0.0001 (two-tailed Mann–Whitney *U*-test). (D) Control and auxin-treated cells were treated with nocodazole for 1 h and fixed with formaldehyde. (E) Experimental timeline for CENP-I depletion. (F,G) SMC2-AID/CDK1^as^ cells were transfected either with control siRNA oligo (F) or CENP-I oligo (G). Cells were further treated with ethanol or auxin. The chromosome mass became symmetrical in SMC2-depleted cells when kinetochore binding to microtubules was perturbed. CENP-T signals were enhanced in (i,iii) in order to visualize the position of kinetochores. Non-enhanced images are also shown (ii,iv). Scale bars: 5 µm.

The mitotic spindle in SMC2-depleted cells was bipolar but often twisted or asymmetric (Figs 1E, 2D and 3Aii,Biii,iv; Movie 2). Mitotic spindles and chromosomes in SMC2-depleted cells did not position properly between the two centrosomes, which were visualized through PACT–RFP (PACT is the pericentrin/AKAP450 centrosomal targeting domain; Fig. 3A,B). In order to quantify the relative positions of chromosomes relative to the mitotic spindles, angles between chromosomes and centrosomes in late prometaphase or metaphase cells were measured in SMC2-AID/CDK1^as^ cells expressing PACT–RFP (see Materials and Methods). The angle between the center of mass of the chromosomes and the two centrosomes was between 155–180° in most (18 out of 22) control cells (Fig. 3B,C). In contrast, this angle was less than 90° in nearly half of the SMC2-depleted cells (13 out of 27).

Forces exerted by spindle attachments exacerbated the structural defects of mitotic chromosomes and altered the relative position and shape of the chromosomes and spindle. When microtubules were depolymerized by nocodazole treatment in control cells, rod-shaped individual mitotic chromosomes could be recognized (Fig. 3D). In contrast, individual chromosomes could not be resolved in SMC2-depleted cells treated with nocodazole although the chromosome mass became more symmetrical and protruding chromatin fibers disappeared.

CENP-I is a kinetochore protein whose depletion by means of RNAi causes defects in kinetochore assembly, visualized as decreased CENP-T levels at kinetochores (Fig. 3E–G). Microtubules do not bind tightly to such compromised kinetochores (Nishihashi et al., 2002; Hori et al., 2008a). In CENP-I-depleted cells, the morphologies of SMC2-depleted chromosomes were still aberrant; however, the chromosome mass was symmetrical and overlapped the mitotic spindle, which also adopted a symmetrical shape (Fig. 3G).

These experiments confirm that condensin is required to shape mitotic chromosomes and reveal that mitotic spindle forces further deform the amorphous SMC2-depleted chromosomes. This is presumably because SMC2-depleted chromosomes lack structural integrity in arms and in pericentromere regions as reported previously (Oliveira et al., 2005; Gerlich et al., 2006; Bouck and Bloom, 2007; Ribeiro et al., 2009) so that pulling forces disrupt the chromosome morphology. This ultimately results in mis-positioning of the chromosome mass and spindle malformations (Wignall et al., 2003; Ono et al., 2004).

### SMC2-depleted kinetochores fail to make stable attachments to microtubules

**Fig. 4.**
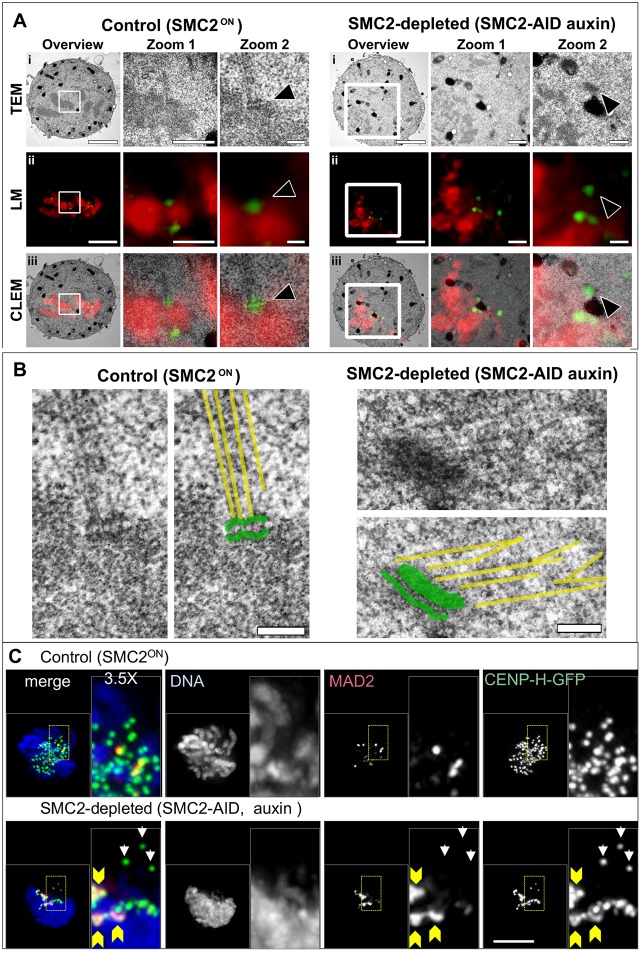
**Compromised kinetochore-microtubule attachments in SMC2-depleted cells.** (A) CLEM of control and SMC2-depleted cells. Images show three progressive magnifications of the white-boxed region, for TEM (i), light microscopy of DAPI (red) and CENP-H–GFP (green) (ii) and a correlative overlay of both the physical and optical sections (iii). The arrowhead in zoom 2 points to a clearly defined trilaminar structure. Scale bars: 3 μm (overview), 1 μm (zoom 1), 200 nm (zoom 2). (B) Enlargement of kinetochores shown in A. Tracings of microtubules (yellow lines) and a kinetochore (green) were overlaid on the TEM pictures, and show the microtubule attachment. Scale bars: 200 nm. (C) SMC2^ON^/CDK1^as^/CENP-H-GFP cells and SMC2-AID/CDK1^as^/CENP-H-GFP cells (treated with auxin) were treated with 1NMPP1 for 4 h and fixed with formaldehyde 1 h after 1NMPP1 washout. Strong MAD2 staining on the centromeres inside of the SMC2-depleted chromosome mass (yellow arrowheads) is not observed on centromeres outside of the chromosome mass (white arrows). Scale bar: 5 µm.

The CENP-H–GFP signals on SMC2-depleted kinetochores that stretched out from the chromosome mass were round (21 out of 21 centromeres) and resembled those in control metaphase cells, where correlative light and electron microscopy (CLEM) revealed a typical trilaminar kinetochore structure with typical end-on microtubule attachment (6 out of 6 and 8 out of 8 kinetochores scored for control and SMC2-AID cells, respectively; Fig. 4A, arrowheads, 4B; Fig. S3A–C). In contrast, the CENP-H–GFP signal on SMC2-depleted kinetochores inside or at the edge of the chromosome mass was often deformed (Fig. 4C, yellow arrowheads, see also Fig. 3Aii and Movie 2). Indeed, the trilaminar structure was less evident following SMC2 depletion (Fig. 4A,B).

The deformed CENP-H–GFP signals mostly corresponded to tight clusters of two or more kinetochores. This is consistent with the observation that we typically resolve far fewer than the expected 80 kinetochores in SMC2-depleted cells. A requirement for condensin to resolve paired kinetochores has been reported in *S. pombe* (Kakui et al., 2017). In addition to kinetochore clusters, we also observed stretched CENP-H–GFP signals (Fig. 3Aii; Movie 2). The shape of kinetochores may be distorted due to lack of structural integrity of the underlying chromatin as previously suggested (Samoshkin et al., 2009). Such defective kinetochores might be less likely to make productive attachments to microtubules, and therefore remain within the disorganized chromatin mass.

Strong MAD2 signals were seen in almost all SMC2-depleted late prometaphase cells (24 out of 25 cells at 1 h after release from 4 h 1NMPP1 block) (Fig. 4C). In contrast, only half of control late prometaphase cells showed strong MAD2 signals (13 out of 25 cells) with the remainder showing only weak MAD2 signals. We hypothesized that these MAD2-positive centromeres lack stable bipolar attachments and that the prometaphase accumulation of SMC2-depleted cells (Fig. 2C) might be due to an unsatisfied spindle assembly checkpoint (SAC).

Indeed, silencing of the SAC by use of MAD2 siRNA caused both SMC2-depleted and control cells to enter anaphase with unaligned chromosomes. Mitotic exit was confirmed by INCENP staining on the central spindle and by reduced cyclin B2 staining (Fig. S4A,Bi,iii,iv). Approximately 80% of SMC2-depleted cells treated with MAD2 siRNA showed highly uneven chromosome segregation with chromosome bridges and centromeres trapped in the intercellular bridge (Fig. S4Aiv,Biv,D). Live-cell imaging showed that all SMC2-depleted daughter cells treated with MAD2 siRNA fused after an abortive cytokinesis (Fig. S4E, Movies 3–6).

Thus, after rapid SMC2 depletion, kinetochores can attach to microtubules but are defective in forming stable amphitelic attachments.

### SMC2 is required for shaping mitotic chromosomes but not for mitotic chromatin compaction

To better understand the consequences of rapid SMC2 depletion on chromosome morphology, we performed an ultrastructural analysis with 3D-EM using serial block face scanning electron microscopy (SBF-SEM) and digital reconstructions (Booth et al., 2016).

**Fig. 5.**
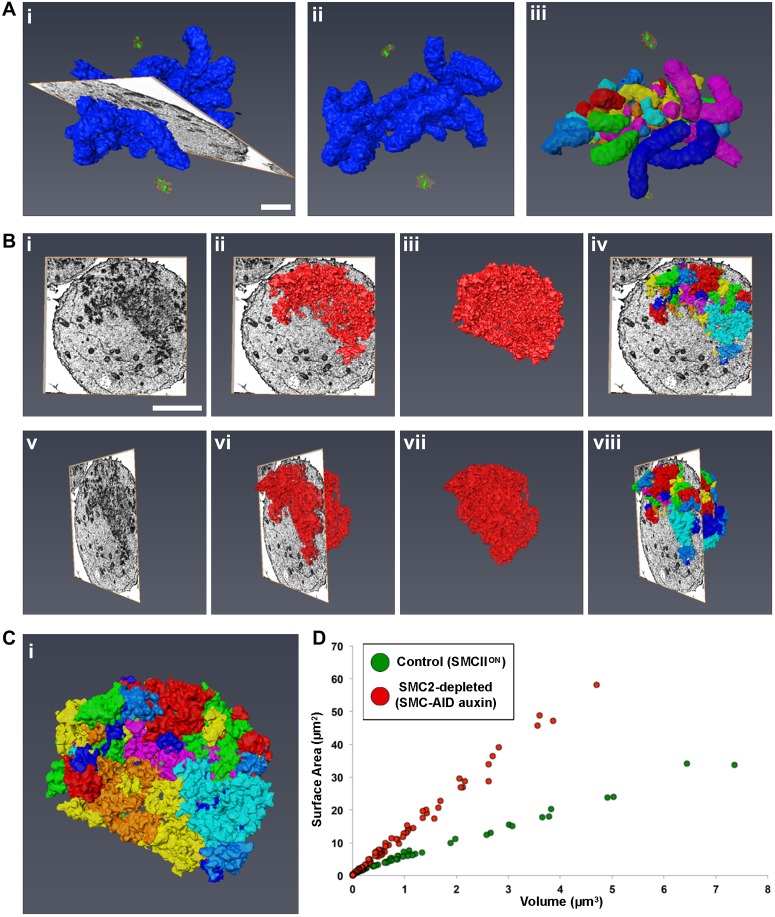
**3D-EM shows that condensin is required for chromosome organization, but not for chromatin compaction.** Control (SMC2^ON^) and condensin-depleted (SMC-AID+auxin) cells were imaged by SBF-SEM. EM data were used to generate digital models in Amira. (A) Representative images of a control cell with all chromosomes (indigo) modeled. Model is shown with (i) and without (ii) the EM orthoslice. Centrioles (green) and pericentriolar material (orange) are also shown. Individual chromosome units were identified (iii) and separated (multi-colored). (B) Representative images of a SMC2-depleted cell with all chromosomes modeled (red), shown at different angles, with and without the EM orthoslice (i–iii,v–vii). Individual chromosome units were identified and separated. Images show chromosomes traversing an orthoslice (iv and viii). (C) Enlargement of the model from Biv. (D) 2D scatter plot of surface area versus volume for all separated chromosome units of control (green) and SMC2-depleted (red) cells. Scale bars: 2 µm (A), 4 µm (B).

To establish a default set of control parameters, the entire chromosome complement of a metaphase SMC2^ON^ DT40 control cell, was reconstructed from 140 serial sections and modeled using Amira (Fig. 5A). The presence of two pairs of centrioles surrounded by PCM and flanking the aligned chromosomes confirmed that the cell was indeed in metaphase. By using separation algorithms, we identified 74 discrete chromosome units (Fig. 5Ai–iii), with a combined volume of 75 µm^3^ and surface area of 424 µm^2^ (Fig. 5D). The normal DT40 karyotype has 80 chromosomes, though scoring of the smallest microchromosomes can be difficult (Sonoda et al., 1998; Molnar et al., 2014).

A similar analysis was next performed on a cell following rapid SMC2 depletion (Fig. 5B). Remarkably, despite the clear disorganization of the chromatin mass, it was possible to resolve 84 chromosome units (Fig. 5C), with a combined volume of 71 µm^3^ and surface area of 931 µm^2^ (Fig. 5D). These segmented units are likely to mostly correspond to individual chromosomes, but may also contain chromosome fragments connected by decondensed regions not detected by our thresholding and segmentation algorithms. Interestingly, although both the chromosome number and combined volumes were similar (74 versus 84 µm^3^ and 75 versus 71 µm^3^), the surface area had more than doubled following SMC2 depletion (424 µm^2^ versus 931 µm^2^). Examining the ten largest (by volume) segmented chromosomes in control and SMC2-depleted cells, revealed that the chromosome shape was highly aberrant in the absence of condensin and remarkably regular in its presence. Mitotic chromatin lacking condensin was compacted (as defined by its volume), but its surface was highly irregular – presumably explaining the increase in surface area recognized by Amira (Fig. S5).

This analysis confirms that, as previously suggested (Vagnarelli et al., 2006), condensin has a key role in establishing a normal mitotic chromosome architecture, but is not essential for mitotic chromatin compaction (as defined by the total volume occupied by the chromatin) from interphase to mitosis.

### Condensin is essential for normal intrinsic architecture of condensed mitotic chromosomes

**Fig. 6.**
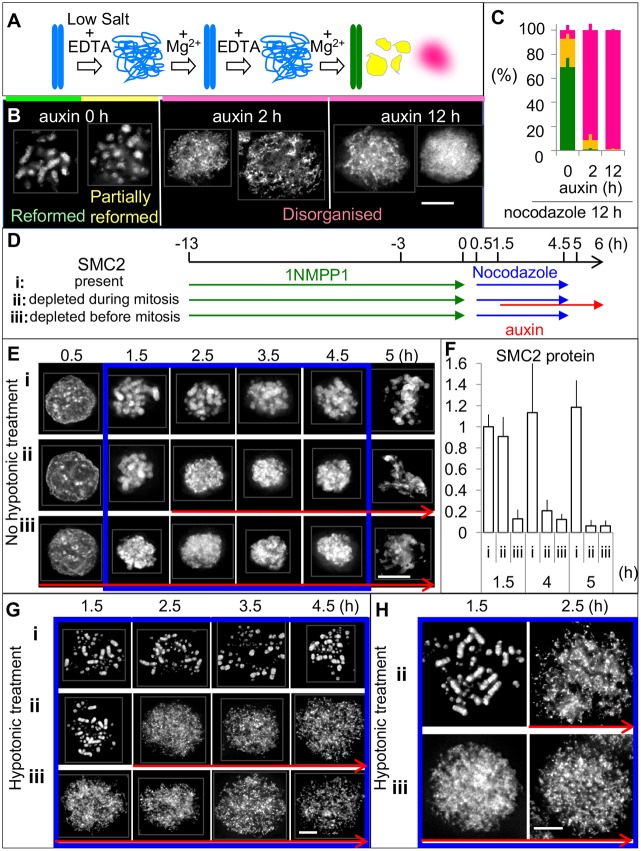
**SMC2 is required to maintain the structure of mitotic chromosomes.** (A) The IMCS assay. Cells were blocked in mitosis with nocodazole. Removal of cations in hypotonic buffer induced the unfolding of chromatin to the level of 10 nm fibers. Addition of Mg^2+^ triggered the shrinking and refolding of chromatin. The shape of chromosomes after two cycles of unfolding and folding was classified as reformed (green), partially reformed (yellow) or disorganized (magenta). (B) Representative images of DAPI-stained mitotic chromosomes at the end of the assay. Scale bar: 5 µm. (C) Quantification of chromosomes at the end of the assay. >50 cells from each individual experiment were counted. Data are plotted as mean±s.d. (*n*=3). (D) Experimental procedure for determining the role of condensin in establishing and maintaining mitotic chromosome structure. Cultures were treated with 1NMPP1 for 13 h. After 1NMPP1 washout, cultures were treated with nocodazole for 4 h to hold the cells in mitosis (*t*=0.5–4.5 h). The mitotic index was reached ∼80% after 1 h of nocodazole addition and did not change until nocodazole washout at 4.5 h. Cells were fixed with formaldehyde at the indicated time points and stained with DAPI before >100 cells were counted for each time point of individual treatments. (E) Cells were fixed with formaldehyde without hypotonic treatment at the indicated time points and stained with DAPI. (F) The amount of SMC2 remaining was measured by immunoblot analysis and normalized to the level of α-tubulin. Data are plotted as mean±s.d. (*n*=3). (G,H) Cells were treated with 75 mM KCl at 37°C for 5 min prior to ice-cold methanol/acetic acid fixation and DAPI staining. Red arrows and blue boxes in E, G and H match treatments as shown in D.

Use of the intrinsic mitotic chromosome structure (IMCS) assay previously established that condensin is required to establish a normal mitotic chromosome architecture (Hudson et al., 2003) (Fig. 6A). In this assay, cells were treated with nocodazole for 12 h before exposure to a hypotonic buffer (Fig. S4F). To test the role of condensin in establishing or maintaining the structural memory of mitotic chromosomes, respectively, cells were treated with auxin either for the entire 12 h (SMC2 depleted before mitotic chromosome assembly) or for the last 2 h (SMC2 depleted after mitotic chromosome assembly). Auxin was omitted from controls so that SMC2–mAID–GFP was continuously present.

For the IMCS assay, chromosomes were subjected to two cycles of unfolding and refolding, then their shape was scored in one of three categories: reformed, partially reformed or disorganized (Fig. 6A,B). Approximately 90% of control cells (without auxin) showed either reformed or partially reformed chromosomes (Fig. 6B,C). In contrast, cells depleted of SMC2 prior to chromosome assembly (auxin for 12 h) exhibited only disorganized chromatin after this treatment. Remarkably, a similar result was obtained for >90% of cells depleted of SMC2 only after mitotic chromosome formation (auxin for 2 h). Depletion of SMC2–mAID–GFP after auxin addition was confirmed by immunoblot analysis (Fig. S4G,H). Thus, the structural memory of mitotic chromosomes is not maintained in the absence of condensin, even when SMC2 is degraded after mitotic chromosomes have formed.

We next exploited the ability of SMC2-AID/CDK1^as^ cells to enter mitosis in a synchronous wave in order to follow the process of chromosome formation in the presence or absence of condensin. We also characterized chromosomes depleted of SMC2 during mitosis (Fig. 6D; Fig. S6).

All cells in the culture were in interphase at the end of a 13 h 1NMPP1 treatment (*t*≈0.5 h) (Fig. 6E). Approximately 80% of those cells entered mitosis within 1 h after 1NMPP1 washout (*t*=1.5 h). The mitotic index then remained almost unchanged until nocodazole washout (*t*=4.5 h).

Mitotic chromosomes were readily visible in control cells during nocodazole treatment (Fig. 6Ei,Gi: *t*=1.5–4.5 h). Chromosomes began to align at a metaphase plate by 30 min after nocodazole washout at *t*=4.5 h (Fig. 6Ei). Most of these cells exit mitosis at *t*=≤6 h, as revealed by a reduction in Cyclin B2 levels (Fig. S6C,Ei,Fi).

Depletion of SMC2 after mitotic chromosome formation revealed that condensin is required to maintain chromosome morphology in mitosis. Rod-shaped chromosomes were clearly visible prior to auxin addition at *t*=1.5 h, but became much less distinct at *t*=2.5 h, within 1 h of auxin addition (Fig. 6Eii). After hypotonic treatment, chromosomes appeared normal at *t*=1.5 h, but were completely disorganized within 1 h of auxin addition at *t*=2.5 h (Fig. 6Gii,Hii).

For cells in which SMC2 had been depleted prior to mitotic entry, mitotic chromosome structure was defective at all time points following release from the 1NMPP1 block (*t*=1.5–4.5 h; Fig. 6Eiii). Without hypotonic treatment, these SMC2-depleted mitotic chromosomes appeared as a condensed ball. In cells treated with hypotonic buffer prior to fixation, SMC2-depleted mitotic chromosomes formed a disorganized mass of dispersed chromatin at all time points (Fig. 6Giii,Hiii).

The morphology of SMC2-depleted mitotic chromosomes and subsequent chromosome mis-segregation defects were indistinguishable regardless of whether SMC2 was depleted prior to mitotic entry or during mitosis after chromosomes had formed. This confirms, for somatic cells, the early observation (Hirano and Mitchison, 1994) and recent studies with mouse oocytes and *Drosophila* embryo (Houlard et al., 2015; Piskadlo et al., 2017) showing that condensin is necessary to maintain the structure of mitotic chromosomes throughout mitosis.

### Titration of condensin levels reveals differential requirements for SMC2 in mitotic chromosome formation and chromosome segregation

Condensin is required both for maintenance of chromosome architecture and for sister chromatid segregation at anaphase. To further explore the functional link between these two processes, we used auxin treatments to produce cultures with a graded series of condensin concentrations. This was possible because the abundance of an AID-tagged protein can be manipulated by varying the auxin concentration (Nishimura et al., 2009).

**Fig. 7.**
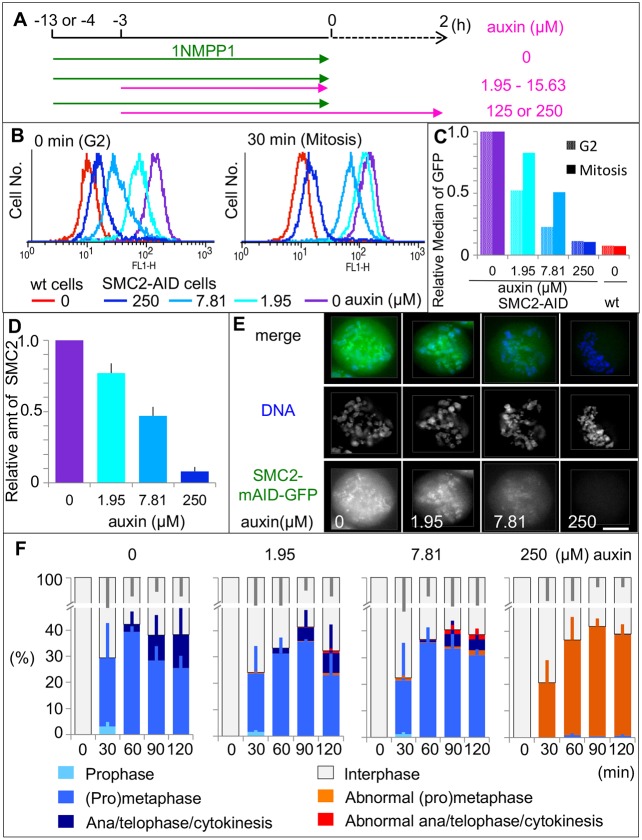
**Cells partially depleted of SMC2 show chromosome mis-segregation.** (A) Experimental timeline for auxin treatment. Auxin was omitted from the medium used for the wash and release except in the case of cells treated with 125 or 250 µM auxin. (B,C) Flow cytometry analysis of SMC2-AID/CDK1^as^ cells. Cells were analyzed at the end of 13 h 1NMPP1 treatment (0 min, G2) and 30 min after washout of 1NMPP1 (30 min, mitosis) with various concentrations of auxin treatment followed by washout. The relative median of the GFP signal is shown (C). (D) The amount of SMC2 remaining in asynchronous culture upon 4 h auxin treatment was measured by immunoblot analysis and normalized to the level of α-tubulin. Data are plotted as mean±s.d. (*n*=3). (E) Representative images of SMC2-AID/CDK1^as^ cells at prometaphase after a 4 h treatment with various concentrations of auxin. Scale bar: 5 µm. (F) Effect of various auxin concentrations on mitotic progression of SMC2-AID/CDK1^as^ cells. After release from 4 h 1NMPP1 treatment, cells were fixed every 30 min. >200 cells were counted for each time point of individual treatments. Data are plotted as mean±s.d. (*n*=3).

Auxin concentrations yielding a range of partial depletions of SMC2–mAID–GFP were determined based on data obtained from flow cytometry, microscopy and immunoblotting analysis (Fig. 7A–E). Depletion levels detected by either flow cytometry or immunoblotting analysis were comparable (Fig. 7C,D). The cell-to-cell variability in this system as measured by flow cytometry analysis was relatively small (Fig. 7B). SMC2-AID/CDK1^as^ cells were treated with 1NMPP1 for 4 h and with various concentrations of auxin for 3 h. After release from 1NMPP1 to allow entry into mitosis, cells were fixed every 30 min and analyzed as in Fig. 2. Auxin was omitted from wash and release media apart from for 125- and 250 µM auxin-treated cells (Fig. 7A) because we noticed that in mitotic cells even low auxin concentrations eventually resulted in depletion levels equivalent to those seen with 125 or 250 µM auxin treatment. This is likely due to the reduction of protein synthesis during mitosis, such that even a low degradation activity will eventually deplete the AID-tagged protein.

Immunoblot analysis showed that the cellular levels of SMC2 fell to ∼77% or ∼47% of those in wild-type cells after addition of 1.95 µM or 7.81 µM of auxin, respectively (Fig. 7D). (The absolute levels vary between experiments but the relative levels are preserved and were independent of the antibody concentration used.) Reduction of SMC2 by 25% had little impact on either the morphology or segregation of mitotic chromosomes (auxin 1.95 µM) (Fig. 7F). However, chromosome segregation defects were observed when SMC2 fell to 50% of wild-type levels (auxin 7.81 µM). In those cells, prometaphase chromosomes were slightly wider, but individual rod-shaped chromosomes were still discernible. Despite this near-normal morphology, some chromatin bridges were observed when the cells entered anaphase and telophase.

**Fig. 8.**
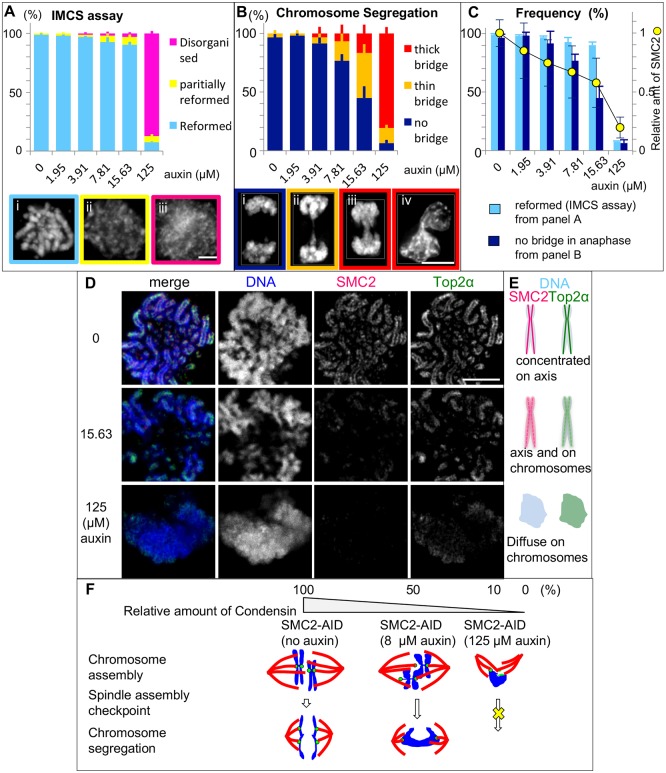
**Effects of lowering condensin levels on chromosome structure and segregation.** Experiments were performed as in Fig. 7A with 1NMPP1 treatment for 4 h (A–C) or 13 h (D). (A) 45 min after 1NMPP1 washout, cells were subjected to the IMCS assay. Representative images are shown. >100 cells were counted for each treatment. Data are plotted as mean±s.d. (*n*=3). (B) 90 min after 1NMPP1 washout, cells were fixed and stained with anti-α-tubulin antibody and DAPI. Representative images are shown. >20 anaphase or telophase cells were counted for each treatment. Cultures treated with 125 µM auxin did not show anaphase/telophase cells, so cells in early cytokinesis (iv) were analyzed. Data are plotted as mean±s.d. (*n*=3). (C) Overlay of key phenotypes from the experiments shown in B,C plotted against the residual levels of SMC2–mAID–GFP as measured by immunoblotting and normalized to the level of α-tubulin in cells treated with the indicated concentration of auxin. (D) 30 min after 1NMPP1 washout, cells were collected, rinsed with PBS and fixed with cold methanol/acetic acid and stained with antibodies for SMC2 and topoisomerase IIα. (E) Diagram summarizing the changes in protein distribution seen in D. (F) Summary of the effects of differing condensin levels on mitotic DT40 cells. DNA (blue), centromeres (green), mitotic spindle (red). Scale bars: 5 µm.

We next asked whether the observed defects in sister chromatid segregation were due to defects in the intrinsic structure of the chromosomes or to another function of condensin. We exposed SMC2-AID/CDK1^as^ cells to 1NMPP1 for 4 h plus various concentrations of auxin for 3 h, then washed out the 1NMPP1 to allow cells to enter mitosis (Fig. 7A). At 45 min after 1NMPP1 washout (when cells were in prometaphase), cells were subjected to the IMCS assay as in Fig. 6A (Fig. 8A). Later, at 90 min after 1NMPP1 washout (when cells were in anaphase or telophase), cells were fixed and stained in order to examine sister chromatid segregation (Fig. 8B). For cells treated with 125 µM auxin, we did not observe typical anaphase or telophase cells, so we instead scored chromatin bridges at cytokinesis.

This experiment revealed very clear differences in the threshold requirements for condensin in chromosome architecture and sister chromatid segregation. In the IMCS assay, mitotic chromosome structures reformed in 90% of cells when SMC2 protein levels had fallen to 60% of wild-type (15.63 µM auxin; Fig. 8A,C). In contrast, only 45% of cells successfully segregated chromosomes without chromosome bridges under these conditions (Fig. 8B,C).

Collectively, these results reveal that higher levels of condensin are required for sister chromatid resolution and anaphase chromosome segregation than for formation of a normal mitotic chromosome architecture, as assayed by the IMCS assay.

### Partial depletion of condensin disrupts the chromosome axes

Use of the auxin titration series revealed that even under conditions of partial condensin depletion where the IMCS assay reports that chromosome architecture is near normal, the organization of the chromosome axes is strongly affected. When condensin levels have been depleted by as little as 40% (15.63 µM auxin), the distribution of condensin and topo IIα is disrupted and the proteins appear to diffuse throughout the chromosomes. At higher auxin concentrations (125 µM), condensin is effectively gone and topoisomerase IIα is no longer visible on the chromatid axes (Fig. 8D,E). Diffuse chromosomal localization of topoisomerase IIα has been reported previously following conventional condensin depletion (Hudson et al., 2003; Coelho et al., 2003). The anaphase bridges observed under partial depletion of SMC2 could be explained by incomplete decatenation and/or possibly recatenation of sister chromatids due to disruption of the chromosome axis and diffuse localization of topoisomerase IIα as recently reported (Piskadlo et al., 2017). Furthermore, this could explain why chromosome segregation defects comprise the most prominent and universal defect when condensin depletion occurs with slower kinetics.

## DISCUSSION

Mitotic chromosome condensation is a complex process, involving removal of interphase chromatin structures (topologically associating domains and compartments), 2–3-fold compaction of the chromatin volume, formation of rod-shaped structures and sister chromatid resolution (Vagnarelli, 2012; Naumova et al., 2013). The role(s) of condensin in these processes remain unclear.

The present study combined use of auxin-inducible degron (AID) technology for rapid condensin depletion with CDK1^as^ alleles to obtain synchronous mitotic entry. Use of SMC2-AID/CDK1^as^ cells allowed us to obtain >10^8^ cells with ≥80% of these being in G_2_, prophase and prometaphase without the use of microtubule inhibitors. We could distinguish the roles of condensin at mitotic entry, during metaphase and during mitotic exit because SMC2 could be depleted just prior to mitotic entry and also within mitosis in a time-restricted manner. Importantly, mitotic chromosomes depleted of condensin after their assembly were morphologically and structurally indistinguishable from chromosomes assembled in the absence of condensin. This is consistent with observations in the *Xenopus* egg extract system (Hirano and Mitchison, 1994) and with a recent study using *Drosophila* embryos (Piskadlo et al., 2017). We speculate that one essential role of condensin is to ‘lock-in’ the architecture of mitotic chromosomes and prevent other factors, such as topoisomerase IIα, from promoting aggregation or random (de)catenation of the chromatin.

Two recent studies have shown that meiotic chromosomes in mouse oocytes and mitotic chromosomes in early *Drosophila* embryos rapidly depleted of condensin lose structural integrity and are morphologically aberrant (Houlard et al., 2015; Piskadlo et al., 2017). Importantly, in both of these studies and in the present work, the resulting chromatin did not look like interphase, but was highly compacted, although the usual rod-like morphology was lost. The three studies thus clearly show that condensin is essential for shaping and maintaining individual mitotic chromosomes *in vivo* in embryos and in somatic cells. These results are consistent with the original studies with *Xenopus* egg extracts and *S. pombe* (Hirano and Mitchison, 1994; Saka et al., 1994). Thus, this role of condensin is evolutionally conserved.

In cells rapidly depleted of SMC2, individual chromosomes could not be distinguished by conventional fluorescence microscopy within the mass of mitotic chromatin, which by conventional electron microscopy resembled a shattered mass of condensed chromatin. However, ultra-structural analysis using 3D-EM with SBF-SEM and digital reconstructions (Booth et al., 2016) resolved the chromosome mass into units that likely correspond to individual chromosomes. Remarkably, the total volume of the entire complement of SMC2-depleted chromosomes was similar to that of control metaphase chromosomes even though the surface area was >4-fold greater.

This result confirmed our previous hypothesis that condensin is dispensable for mitotic chromatin volume compaction, but is required for structural organization of the condensed chromosomes (Vagnarelli et al., 2006; Samejima et al., 2012). Surprisingly, mitotic chromosomes can still form after conventional depletion of all three major components of the mitotic chromosome scaffold (condensin, topoisomerase IIα and KIF4A) (Samejima et al., 2012). Furthermore, extensive proteomic studies failed to identify other major structural components of mitotic chromosomes likely to function in promoting mitotic chromatin compaction (Ohta et al., 2010; Samejima et al., 2015). It is likely that chromatin volume compaction in mitosis is mediated by factors intrinsic to the chromatin, possibly including histone post-translational modifications (Markaki et al., 2009; Georgatos et al., 2009; Kruitwagen et al., 2015; Zhiteneva et al., 2017).

In addition to obvious effects on chromosome architecture, rapid depletion of SMC2 led to spindle defects, including exclusion of the chromosomes from an abnormally shaped mitotic spindle. One characteristic of condensin depletion is the presence of thin chromatin fibers that stretch out from the bulk of the chromatin and end in a centromere. The stretched fibers correspond to centromeric heterochromatin that becomes abnormally compliant after condensin depletion and is stretched by spindle forces acting on kinetochores (Ribeiro et al., 2009; Samoshkin et al., 2009; Jaqaman et al., 2010). Interfering with microtubule attachments at kinetochores eliminates these thin fibers and the chromosomes form a more spherical mass.

It has long been known that tension stabilizes kinetochore–microtubule attachments (Nicklas and Koch, 1969; Akiyoshi et al., 2010; Miller et al., 2016). Thus, the lack of tension, compromised kinetochore structure or possibly mislocalized aurora B kinase activity due to condensin loss could affect kinetochore–microtubule attachments. We speculate that force on the kinetochores from both the mitotic spindle and the bulky chromosome mass causes the incorrect centromere positioning relative to the spindle. This may result in the abnormal mitotic spindle morphology observed in SMC2-depleted cells and possibly promote incorrect kinetochore–microtubule attachments (Saunders and Hoyt, 1992; Wignall et al., 2003; Ono et al., 2004).

The availability of an AID degron allele allowed us to examine the effects of creating a hypomorphic series in which levels of SMC2 were lowered to differing extents (Nishimura et al., 2009). Use of the titration series revealed that higher condensin levels are required for successful anaphase sister chromatid separation than for establishing the metaphase chromosome architecture, as defined by our IMCS assay. This could explain why severe mitotic chromosome assembly defects were not reported in experimental systems with gradual depletion of condensin. Interestingly, SMC2-AID/CDK1^as^ cells uniformly showed more-severe defects than those seen in SMC2^OFF^ conditional knockout cells although residual SMC2 was similar in both systems (5–7% of wild-type levels). Thus, the time taken to deplete the protein appears to be a critical determinant of the resulting phenotype.

Why is the threshold amount of SMC2 higher for segregating chromosomes? Partial condensin depletion appears to disrupt the orderly distribution of condensin and topoisomerase IIα along the chromatid axes. This altered distribution of the two proteins could potentially cause anaphase chromosomes to become mechanically more fragile or interfere with sister chromatid decatenation. It will be interesting in the future to explore whether the different threshold levels of condensin for chromosome condensation and sister chromatid separation reflect different functions of the condensin I and II complexes in these two processes (Sullivan et al., 2004; D'Amours et al., 2004). In other experiments, cells rapidly depleted of either condensin I or II complex alone form morphologically recognizable, but distinct, mitotic chromosomes and undergo anaphase/telophase with chromosome segregation defects (Gibcus et al., 2018).

In summary, we show here that mitotic chromosome architecture in somatic cells is not static, but must be maintained by condensin acting throughout mitosis. In the absence of condensin, chromatin is fully compacted in volume but lacks a normal architecture. The role of condensin in shaping mitotic chromosomes is essential for proper kinetochore–microtubule attachments and also for correct shaping and positioning of the mitotic spindle. Wild-type levels of condensin are particularly critical to ensure the completion of sister chromatid separation in anaphase even though the bulk of sister chromatid resolution normally occurs during prophase (Nagasaka et al., 2016). Taken together, these studies highlight the importance of rapid depletion strategies in synchronized cells when determining the phenotypic consequences of eliminating important molecular machines like condensin.

## MATERIALS AND METHODS

### Cell culture, transfection and siRNA

Chicken DT40 (B lymphoma) cells were cultured in RPMI1640 medium supplemented with 10% fetal bovine serum and 1% chicken serum at 39°C in 5% CO_2_ in air. All the cell lines used for this study were grown in the absence of antibiotics and tested negative for mycoplasma contamination.

Stable transfection of DT40 cells was performed as described previously (Samejima et al., 2008). We utilized the Neon setting 5 (ThermoFisher Scientific) to transiently transfect plasmid DNA (4–10 µg) or siRNA oligonucleotides (2 µl of 100 µM) into 2×10^6^–4×10^6^ cells suspended in 100 µl buffer R from the Neon kit. MAD2 siRNA duplex oligonucleotides with sequence 5′-GAAAGCCAUUCAGGAUGAAAUUCGA-3′ and 5′-UCGAAUUUCAUCCUGAAUGGCUUUC-3′ (ThermoFisher Scientific) were synthesized. Control oligonucleotide (AllStars Negative Control siRNA) was purchased from QIAGEN.

### Cell lines

SMC2 conditional DT40 knockout (SMC2^ON/OFF^) cells were previously established (Hudson et al., 2003). In brief, the endogenous *SMC2* gene locus (exon 1–6 including the start codon) was disrupted by a Histidinol-resistance cassette. These cells depend on the expression of a *Gallus gallus* (Gg)*SMC2* cDNA driven by a tetracycline suppressible promoter (SMC2^ON^). Addition of doxycycline stops the expression of SMC2 protein. SMC2 protein was undetectable in cells treated with doxycycline for >30 h (SMC2^OFF^).

SMC2-AID DT40 cells were based on SMC2^OFF^ cells constantly cultured in the presence of doxycycline (0.5 µg/ml) so that non-tagged GgSMC2 protein was not present in the cells. These cells were transfected with a GgSMC2 cDNA fused with a minimal AID (mAID) tag (AtIAA17 amino acids 65–132) (Natsume et al., 2016) and GFP tag (SMC2–mAID–GFP) at the C-terminus, driven by a 3.8 kb fragment of the GgSMC2 promoter (Hudson et al., 2003). SMC2–mAID–GFP was present as the sole source of SMC2 protein in SMC2-AID cells (Fig. 1A). A CMV promoter drove the expression of the plant-specific F-box protein OsTIR1 linked to a *Mus musculus* (Mm)DHFR cDNA by a T2A peptide (synthesized at ThermoFisher Scientific and cloned into pCDNA3). 10 µM methotrexate (MTX) was used to select cells expressing MmDHFR as well as OsTIR1 at a high level. Plasmids encoding SMC2–mAID–GFP and OsTIR1 were randomly integrated into the genome.

CDK1as DT40 cells have a CMV promoter driving the expression of the *Xenopus laevis* (Xl)Cdk1^as^ cDNA (Hochegger et al., 2007) linked to a puromycin-resistance gene by a T2A peptide. The GgCdk1 gene was inactivated by transient transfection of plasmids encoding human (h)Cas9 cDNA (Addgene #41815) and a GgCdk1 guideRNA (based on Addgene #41824) (Mali et al., 2013). The target sequence of the guide RNA was 5′-AAAATACGTCTAGAAAGTG-3′.

Where desired, a CENP-H–GFP-encoding vector (gift of Yasunari Takami, University of Miyazaki, Japan) was used to tag the endogenous CENP-H locus in DT40 cell lines. The PACT–RFP construct was a gift of Viji Draviam (University of Cambridge, UK).

### Antibodies and drug treatments

Antibodies used for immunoblotting and indirect immunofluorescence analysis were: rabbit anti-SMC2 (1:500) (Saitoh et al., 1994), guinea pig anti-GgTopo 2α (1:1000) (Earnshaw et al., 1985), rabbit anti-GgCENP-T (1:1000) (Hori et al., 2008b), and rabbit anti-MAD2 (1:100, gift of Tatsuo Fukagawa, Graduate School of Frontier Biosciences, Osaka University, Japan), rabbit-anti GgcyclinB2 (gift of Eric Nigg, retired from Biozentrum, University of Basel, Switzerland), mouse anti-α-tubulin DM1A (1:1000, lot no. 066M4870V, Sigma-Aldrich), mouse anti-Cdk1 monoclonal A17 (1:200–1:500, lot no. GR133813-4, Abcam), and rabbit anti- phospho-histone H3 (Ser 10) (D2C8) (1:1600, lot no. 3, Cell Signaling Technology).

Nocodazole dissolved in DMSO was added to a final concentration of 0.5 µg/ml (Sigma-Aldrich). Doxycycline dissolved in water was added to a final concentration of 0.5 µg/ml (BD) unless stated otherwise. 1NMPP1 dissolved in DMSO was added to a final concentration of 2 µM. Indole-3-acetic acid (auxin) dissolved in ethanol was added to a final concentration of 125 µM unless stated otherwise (Fluka, Sigma-Aldrich).

### Use of CDK1^as^ system

Typically, CDK1^as^ cells were treated with 2 µM 1NMPP1 for 4 h to accumulate cells in G_2_ phase. 1NMPP1 is a bulky ATP analogue that reversibly and specifically inhibits analogue-sensitive kinase mutants. 1NMPP1 treatment longer than 4 h results in an increase of multipolar spindles due to centrosome amplification during the G_2_ block. However, CDK1^as^ cells blocked at G_2_ phase for more than 10 h could still enter mitosis synchronously after 1NMPP1 washout without any apparent problems apart from the multipolar spindle. In order to release from the 1NMPP1 block, cells were washed two to three times with fresh medium.

### Indirect immunofluorescence of fixed cells

Cells were fixed in pre-warmed 4% formaldehyde in PBS for 10 min and permeabilized with 0.15% Triton X-100 for 5–10 min. Cells were blocked with 5% bovine serum albumin (BSA) in PBS for 30 min. Primary antibody diluted in the blocking buffer was applied to the cells for 1 h. Cells were washed with PBS 3× for 5 min. Secondary antibody diluted in the blocking buffer [1:500–1:1000, Alexa Fluor 488, 555 or 594, 647 (Molecular Probes, ThermoFisher Scientific)] was applied to the cells for 30 min. Cells were washed with PBS for 5 min three times. DNA was stained with Hoechst 33452 and mounted with Prolong diamond (Molecular Probes, ThermoFisher Scientific).

#### DeltaVision microscopy

3D datasets were acquired using a cooled CCD camera (CoolSNAP HQ; Photometrics) on a wide-field microscope (DeltaVision Spectris; Applied Precision) with a 100× NA 1.4 Plan Apochromat lens. The datasets were deconvolved with softWoRx (Applied Precision), converted into Quick Projections in softWoRx, exported as TIFF files, and imported into Adobe Photoshop for final presentation.

#### 3D-SIM

Super-resolution images were acquired using structured illumination microscopy. Samples were prepared on high precision cover-glasses (Zeiss, Germany). 3D SIM images were acquired on an N-SIM (Nikon Instruments, UK) instrument using a 100×1.49 NA lens and refractive index-matched immersion oil (Nikon Instruments). Samples were imaged using a Nikon Plan Apo TIRF objective (NA 1.49, oil immersion) and an Andor DU-897X-5254 camera using 405, 488 and 561 nm laser lines. Step size for *Z*-stacks was set to 0.120 μm as required by the manufacturer's software. For each focal plane, 15 images (five phases, three angles) were captured with the NIS-Elements software. SIM image processing, reconstruction and analysis were carried out using the N-SIM module of the NIS-Element Advanced Research software. Images were checked for artifacts using the SIMcheck software (http://www.micron.ox.ac.uk/software/SIMCheck.php). Images were reconstructed using NiS Elements software (Nikon Instruments) from a *z*-stack comprising at least 1 µm of optical sections. In all SIM image reconstructions the Wiener and Apodization filter parameters were kept constant. 3D datasets were visualized and analyzed using Imaris V8.4 (Bitplane, Oxford Instruments, UK).

#### Zeiss Airyscan microscopy

Images of chromosome scaffold proteins on chromosome spreads were obtained by using the Airyscan module on a Zeiss LSM 880 confocal, using a ×100 alpha Plan-Apochromat objective. Hoechst 33342 was detected using a 405 nm diode laser and a 420–480 nm bandpass emission filter. Alexa Fluor 488 was detected using the 488 nm line of an argon laser and a 495–550 nm bandpass emission filter. Alexa Fluor 555 was detected using a HeNe561 laser and a 570 nm long pass emission filter. Alexa Fluor 647 was detected using a 633 laser and a 605 nm long pass emission filter. Step size for *z*-stacks was set to 0.145 µm. 3D datasets were visualized and analyzed by using Imaris V8.4 software (Bitplane, Oxford Instruments, Oxfordshire, UK).

### Live-cell imaging with Zeiss Airyscan microscopy

Cells were centrifuged and suspended into Leibovitz's L-15 Medium (ThermoFisher Scientific) supplemented with 10% fetal bovine serum (FBS) and 1% chicken serum. DNA was visualized by means of 1 µM SiR DNA (Spirochromome). GFP was detected using the 488 nm line of an argon laser and a 495–550 nm bandpass emission filter, RFP was detected using a HeNe561 laser and a 570 nm long-pass emission filter. SiR DNA was detected using a 633 nm laser and a 605 nm long-pass emission filter. Step size for *z*-stacks was set to 0.4 µm. Images were taken every 5 min. 3D datasets were visualized and analyzed by using Imaris V8.4 software.

### IMCS assay

Cells were enriched in mitosis either by 12 h nocodazole treatment (0.5 µg/ml) (Fig. 6A–C) or by 1NMPP1 treatment (Fig. 8A,B,D). Auxin was added at the indicated time points (Fig. 8A). The cells were plated on polylysine-coated slides for 30 min before the following treatments. The slides were rinsed with PBS, immersed in TEEN buffer for 5 min (1 mM triethanolamine-HCl pH 8.5, 0.2 mM NaEDTA and 25 mM NaCl), before immersion in RSB buffer for 15 min (10 mM Tris-HCl pH 7.4, 10 mM NaCl, and 5 mM MgCl_2_). Following two cycles of TEEN buffer and RBS buffer treatments, the cells were fixed with 4% formaldehyde in RBS buffer and mounted with Vectashield (containing DAPI). The final chromosome morphologies were classified as reformed (green), partially reformed (yellow) or disorganized (magenta).

### Quantitative immunoblotting

Membranes were incubated with the relevant primary antibodies recognizing α-tubulin (as a loading control), SMC2, and MAD2, then subsequently with IRDye-labeled secondary antibodies (LI-COR Biosciences). Fluorescence intensities were determined by using a CCD scanner (Odyssey; LI-COR Biosciences) according to the manufacturer's instructions.

### Flow cytometry analysis

GFP-positive and -negative living cells were analyzed using a FACSCalibur flow cytometer following the manufacturer's instructions.

In order to assess the DNA content of the cells, cells were fixed with ice-cold 70% ethanol overnight. These cells were rinsed with PBS then resuspended in PBS containing 100 µg/ml RNAse A and 5 µg/ml Propidium Iodide and were analyzed using a FACSCalibur flow cytometer following the manufacturer's instructions.

### Measurement of the angle of chromosome mass and centrosomes

SMC2-AID/CDK1^as^ cells expressing PACT–RFP were treated with 1NMPP1 for 4 h with or without a 3 h auxin treatment. At 60 min after 1NMPP1 washout, cells were fixed in pre-warmed 4% formaldehyde in PBS for 10 min. DNA was stained with Hoechst 33452, and cells were mounted with Prolong diamond (Molecular Probes, ThermoFisher Scientific). Pictures of late prometaphase and metaphase cells were taken using the Zeiss Airyscan microscopy as described above. The (*X, Y, Z*) coordinates of the center of homogenous mass of PACT–RFP signals (*X_1_, Y_1_, Z_1_*) and (*X_3_, Y_3_, Z_3_*), and chromosomes (*X_2_, Y_2_, Z_2_*) were obtained by using Imaris V8.4 software (Bitplane, Oxford Instruments, Oxfordshire, UK). The angle between the chromosome mass and centrosomes was calculated based on the coordinates.

### SBF-SEM

#### Preparation of cells

Cells were seeded onto gridded dishes (MatTek) and fixed with 3% glutaraldehyde and 1% paraformaldehyde in 0.1 M sodium cacodylate buffer for 1 h at room temperature. Cells were then washed with PBS three times for 5 min each time and samples prepared for SBF-SEM (West et al., 2010). Extra contrasting steps were introduced compared to those used for standard transmission electron microscopy (TEM) to reduce charging and improve the signal-to-noise ratio. In detail, following fixation, the cells were post fixed and stained with reduced osmium (2% osmium tetroxide in dH_2_O plus 1.5% potassium ferrocyanide in 0.1 M sodium cacodylate buffer) for 1 h at room temperature. This was followed by 0.1% tannic acid in ddH_2_O for 20 min at room temperature. A second osmication step (2% in ddH_2_O for 40 min at room temperature), preceded an overnight incubation in aqueous 1% uranyl acetate at 4°C. The next day cells were stained with Walton's lead aspartate (0.02 M in lead nitrate plus 0.03 M in aspartic acid in ddH_2_O, adjusted to pH 5.5) for 30 min at 60°C. To prevent precipitation artifacts, the cells were washed for a minimum of five times for 3 min each time with ddH_2_O between each of the staining steps described. Next, samples were dehydrated in a graded ethanol series of 30%, 50%, 70%, 90% in ddH_2_O for 5 min each, followed by twice (5 min each) in 100% ethanol. Samples were then infiltrated with TAAB Hard Premix resin at ratios of 1:1, 2:1 and 3:1 resin:100% ethanol, for 30 min per incubation. Finally, samples were incubated in 100% resin twice (30 min each), before embedding the whole dish in 2 mm of 100% fresh resin. Samples were cured for 48 h at 60°C.

#### Preparation of blocks for 3View SBF-SEM

Resin is separated from the gridded dish by trimming away the excess plastic and carefully sliding a razor between the dish and the resin (Booth et al., 2013). Excess resin is removed by using a junior hacksaw and scalpel before the block is mounted onto a cryo-pin, cell side up, using superglue. Targeted trimming is performed using an ultra-microtome and etched coordinates (Booth et al., 2013).

#### SBF-SEM imaging and acquisition

Samples were painted with Electrodag silver paint (avoiding the block face) and then coated with 10 nm AuPd using a Q150T sputter coater (Quorum Technologies). The sample was inserted into the Gatan 3View sample holder and adjusted so the block face would be central in the microtome and parallel with the knife edge. Consecutive sectioning and imaging was performed. To obtain a typical resolution of 12 nm in *x* and *y*, a frame width of 1024×1024 was used. he section thickness was 60 nm over 200–400 sections.

#### 3D reconstruction, modeling and segmentation

3View EM stacks were annotated using Amira (FEI). Chromosomes present in every orthoslice were annotated by using the ‘masking’ and ‘thresholding’ tools alone (fully automated) or in combination with ‘magic wand’ and ‘blow’ tools (semi automated).

The modeled complement of chromosomes was segmented into discernible isolated objects by using the ‘interactive thresholding’ and ‘separate objects’ modules. Objects were separated using 3D interpretation and a neighborhood criteria of 26 connected elements, by at least one corner, edge or face. The marker contrast range (*H-extrema*) was set between 5 and 7, depending on the sample. The ‘label analysis’ modules were used to measure the geometry of all isolated structures. Surface renders were generated using unconstrained smoothing at levels 5–7.

### Correlative light and electron microscopy

The CLEM processing method was an adapted version of a previously established protocol (Booth et al., 2013). Cells were seeded onto glass-bottomed, gridded dishes (MatTek Corporation, USA), and coated with poly-l-lysine (PLL). After 30 min, cells were fixed for 1 h (2% glutaraldehyde, 2% paraformaldehyde in 0.2 M sodium cacodylate buffer containing 5 μg/ml Hoechst 33342) and washed in PBS (three times for 5 min each time). Cells of interest were identified using a wide-field epi-fluorescence microscope (DeltaVision RT; Applied Precision). GFP-expressing mitotic cells were located and their position mapped by using transmitted light to visualize reference coordinates. High-powered images were also acquired. Next, cells were osmicated (1% osmium tetroxide in PBS) for 1 h, washed with PBS (three times for 5 min each time), ddH2O (twice for 20 min each time), and then 30% ethanol (once for 10 min) before contrast staining with uranyl acetate (0.5% in 30% ethanol) for 1 h. Cells were then dehydrated using a graded series of ethanol washes culminating in two 10 min incubations with 100% ethanol, followed by in infiltration with ethanol:resin mixtures (at 2:1 and then 1:1). Finally, cells were embedded in 100% resin, with a gelatin capsule of resin covering the cells of interest, before curing at 60°C for 48–72 h. Ultra-small resin blocks (50 μm^2^) were trimmed and serial sections (85 nm thickness) taken at areas corresponding to previously chosen coordinate positions, before post-staining in Reynold's lead citrate and uranyl acetate (5% in 50% ethanol) for 10 and 5 min, respectively. Images were taken at 100 kV using an FEI Technai G2 Spirit BioTWIN microscope.

The appropriate *z*-position CLEM images were concatenated with ImageJ and overlaid using Photoshop Elements 6 (Adobe). Overlays are shown only as a qualitative tool to help the reader follow structures of interest present in both light microscopy and electron microscopy panels. To account for discrepancies in optical and physical sections, misaligned approach angles and processing artifacts, scaled resizing of light microscopy panels has been performed to generate a cleaner overlay.

### 1NMPP1 analogue synthesis

Synthesis of the ATP analog 4-amino-1-tert-butyl-3-(1′-naphthylmethyl)-pyrazolo[3,4-d]pyrimidine (1NMPP1) was carried out in five steps as described by Hanefeld et al. (1996) with the following modifications. The starting material was 1-naphthaleneacetic acid and the first two steps were carried out under N_2_. In step 2, triethylamine was used in place of NaH and the product 1-naphthaleneacetyl malonylnitrile was recrystallized from acetonitrile and chloroform. The enol ether product of step 3 was purified on a silica gel column (elution with 3:1 hexane:ethyl acetate) and stored under N_2_. The final product, a white solid that gave a single spot via thin-layer chromatography (TLC), was purified similarly and further recrystallized from ethyl acetate. Its identity was confirmed by ^1^H nuclear magnetic resonance (NMR), HH correlation spectroscopy (COSY) and high-resolution mass spectrometry. ^1^H NMR (270 MHz, CDCl_3_) gave peaks at 1.84 (singlet, 9H), 4.75 (singlet, 2H), 4.85 (broad singlet, 2H), 7.18 (doublet, 1H), 7.38 (triplet, 1H), 7.54 (multiplet, 2H), 7.79-7.92 (multiplet, 2H), 8.22 (doublet, 1H) and 8.24 (singlet, 1H), in close agreement with published data (Bishop et al., 1999). Mass spectrometry gave a mass of 331.181712 Da for the molecular ion (calculated mass for C_20_H_21_N_5_, 331.179696 Da). The solid compound was stored at 4°C. Aliquots were dissolved in DMSO at 10 mM and stored at −20°C.

## Supplementary Material

Supplementary information
