# Supplementary material for: Functional analysis after rapid degradation of condensins and 3D-EM reveals chromatin volume is uncoupled from chromosome architecture in mitosis
Source: J Cell Sci. 2018 Feb 15;131(4):jcs210187. doi: 10.1242/jcs.210187 (PMC5868952; doi:10.1242/jcs.210187)
Supplement: Supplementary information [file joces-131-210187-s1.pdf]

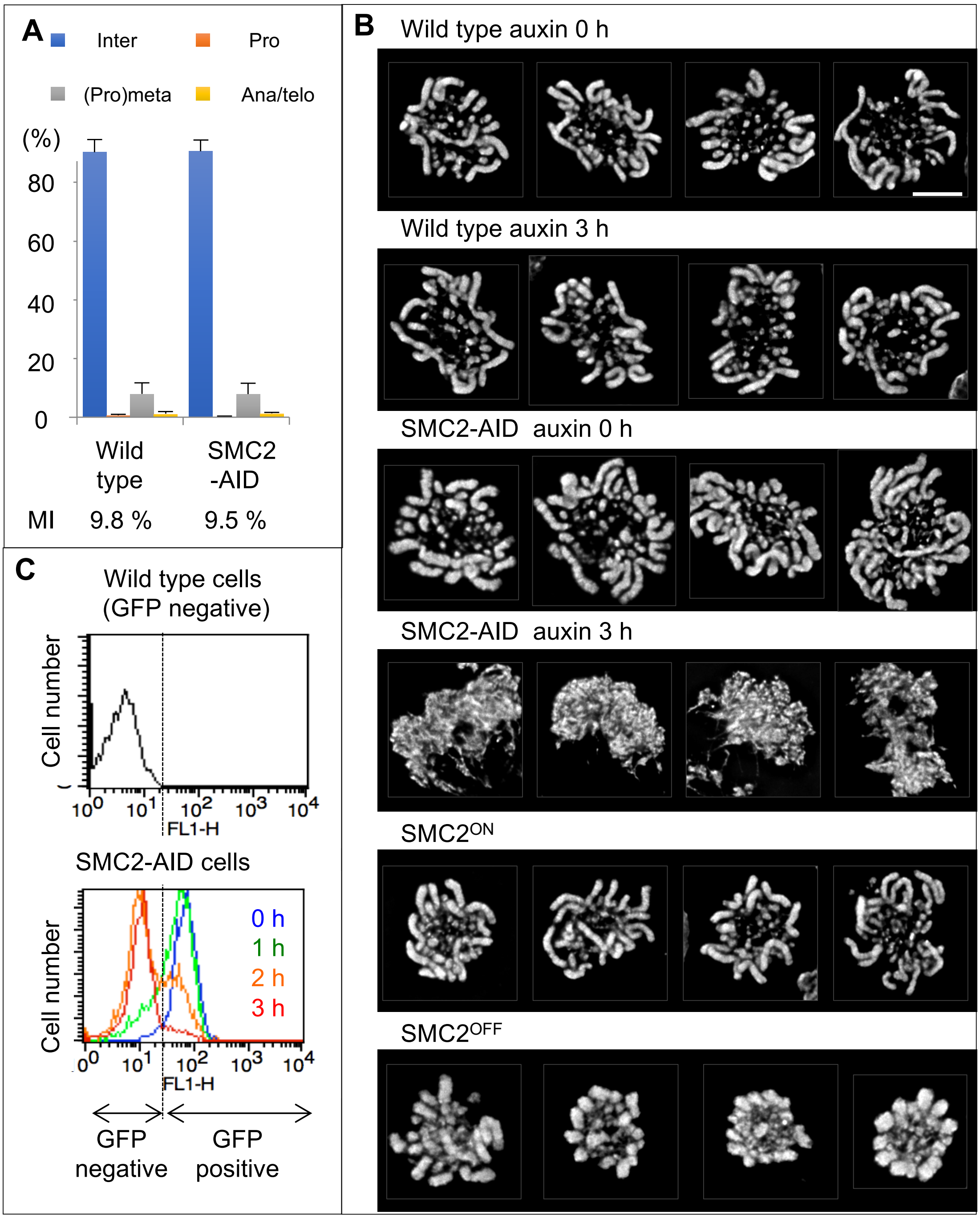

Supplementary Figures:

Fig. S1. Characterisation of SMC2-AID cell line. (A) Asynchronous wild type/CDK1^{as} cells and SMC2-AID/CDK1^{as} cells were fixed with formaldehyde and stained for DNA, α-tubulin, and H3S10ph. 500 cells were counted for each sample. Data plotted as mean ± s.d. (*n*=3). **(B)** Cells were treated as Fig. 1G. Scale bar: 5 µm. **(C)** Flow cytometry analysis of GFP positive cells treated with auxin for 0 - 3 h.

Journal of Cell Science • Supplementary information

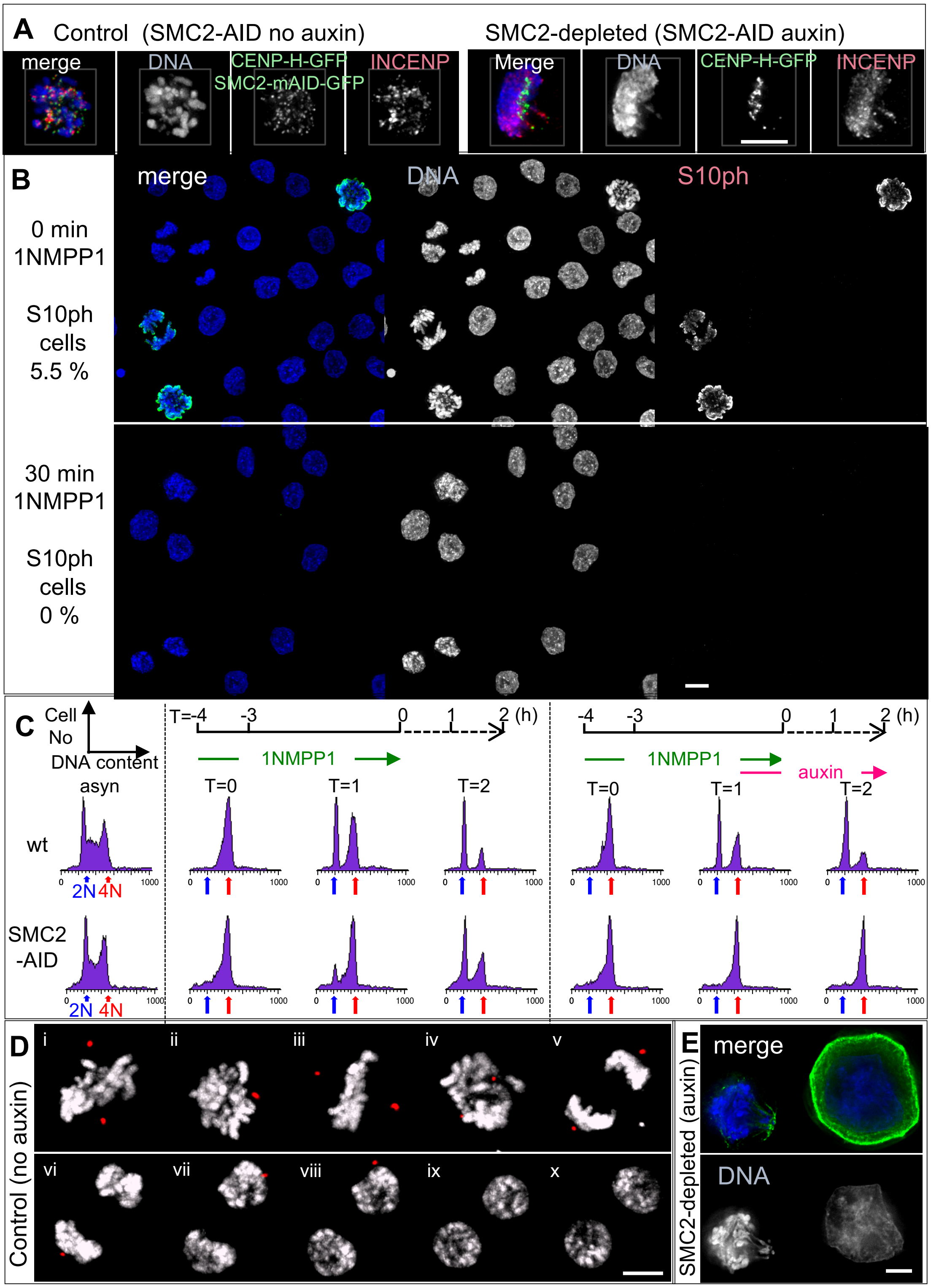

Journal of Cell Science • Supplementary information

Fig. S2. Mitotic cells depleted of SMC2. (A) INCENP (red) localises on chromosomes in early mitosis in SMC2-AID/CDK1^{as}/CENP-H-GFP cells both treated or not treated with auxin. Control (no auxin) and SMC2-depleted (auxin 3 h). **(B)** Asynchronous wild type/CDK1^{as} cells were treated with 1NMPP1 for 0 or 30 min and stained for DNA (blue) and H3S10ph (red). **(C)** Analysis of DNA content of wild type/CDK1^{as} cells (wt) and SMC2-AID/CDK1^{as} cells using flow cytometry. Cells from asynchronously growing culture (i), 4 h block and release from 1NMPP1 (ii), or 4 h block and release from 1NMPP1 with auxin (iii) were analysed as described in Materials and Methods. Positions of cells with 2N DNA (blue arrow) or 4N DNA (red arrow) are shown. **(D)** Stills from live-cell imaging of SMC2-AID/CDK1^{as} cells expressing Pericentrin/AKAP450 centrosomal targeting (PACT)-RFP (control). DNA was stained with SiR-DNA. 3D image stacks were collected every 5 mins at 0.4-µm z increments on a Zeiss Airyscan microscope. SMC2-AID/CDK1^{as} cells exited mitosis (v-x). Stills of an SMC2-depleted cell (auxin) is shown in Fig. 2E. **(E)** SMC2-AID cells treated with auxin for 2 days. Scale bars: 5 µm.

Journal of Cell Science • Supplementary information

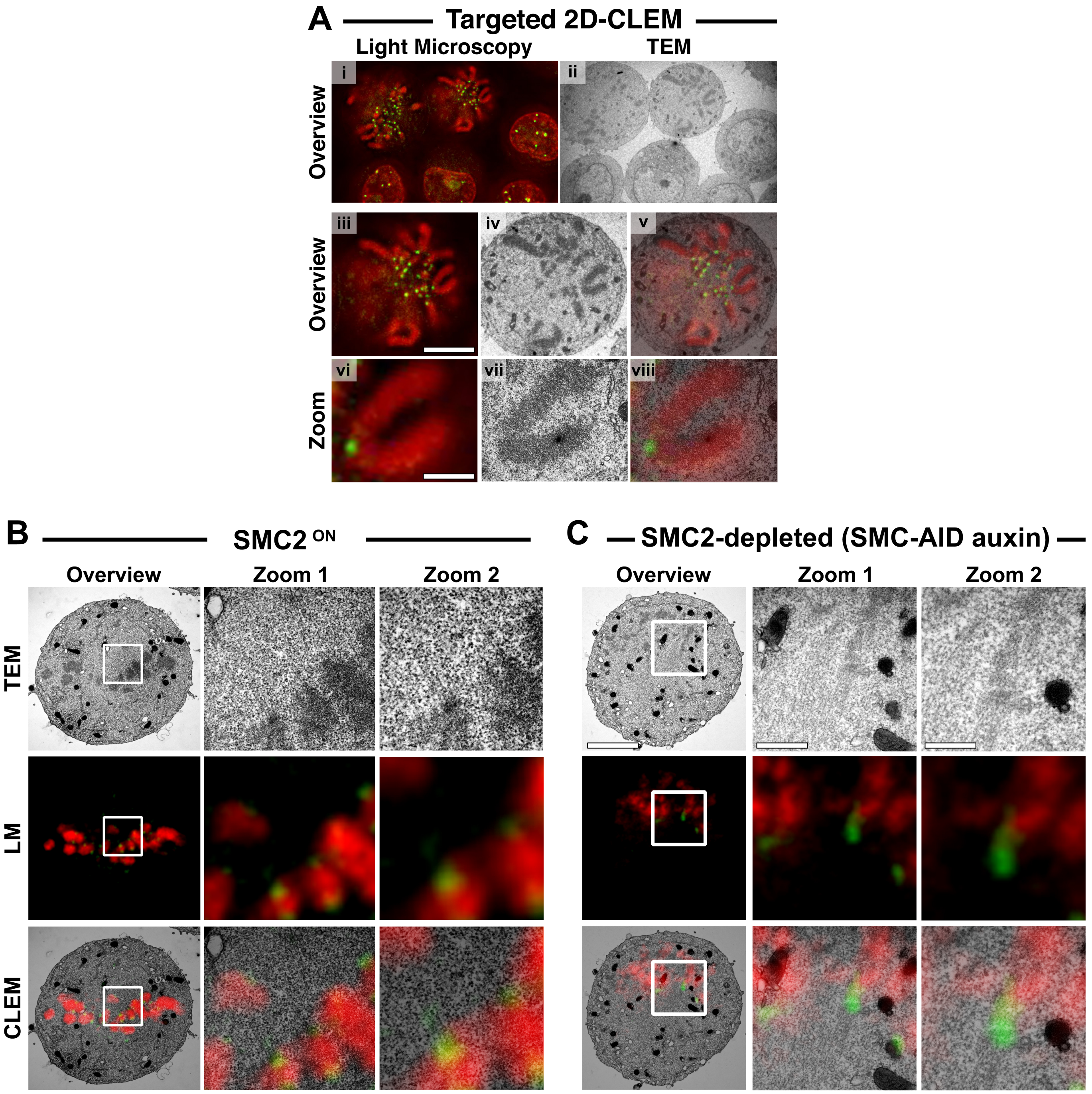

Journal of Cell Science • Supplementary information

Fig. S3. Analysis of kinetochore-microtubule attachments in SMC2-depleted cells. (A) Representative example of the CLEM strategy used to analyse microtubules and chromosomes of DT40 cells and the challenges involved. (i) Light microscopy (DAPI – red, CENP-H-GFP green) is used as a guide to re-find areas of interest by TEM. For example, a chromosome that appears to be resting parallel to the imaging/sectioning plane, and has clear centromeric labeling. (ii) Consecutive 75 nm EM sections are acquired and studied until the appropriate correlative sections are identified. (iii-v) The appropriate cell of interest is confirmed by overlaying (v) the light microscopy (iii) and TEM (iv) images, before the original identified region/structure of interest is re-confirmed using high powered light microscopy (vi) and TEM images (vii), allowing a near perfect overlay (vii). Upon completion of this analysis, no microtubules are visualized interacting with the kinetochore. **(B, C)** Additional supporting data for Fig. 4. Correlative light and transmission electron microscopy of SMC2^{ON} (control) and SMC2-depleted cells (SMC-AID auxin). Images show two progressive zooms (Zoom 1, Zoom 2) of white-boxed regions in the overview for TEM, LM (DAPI – red, CENP-H-GFP - green) and a correlative overlay of the two (CLEM). Scale Bars: 10 µm (Ai), 3 µm (Aiii), 500 nm (Avi), 3 µm (C overview), 500nm (C zoom 1) and 200 nm (C zoom 2).

Journal of Cell Science • Supplementary information

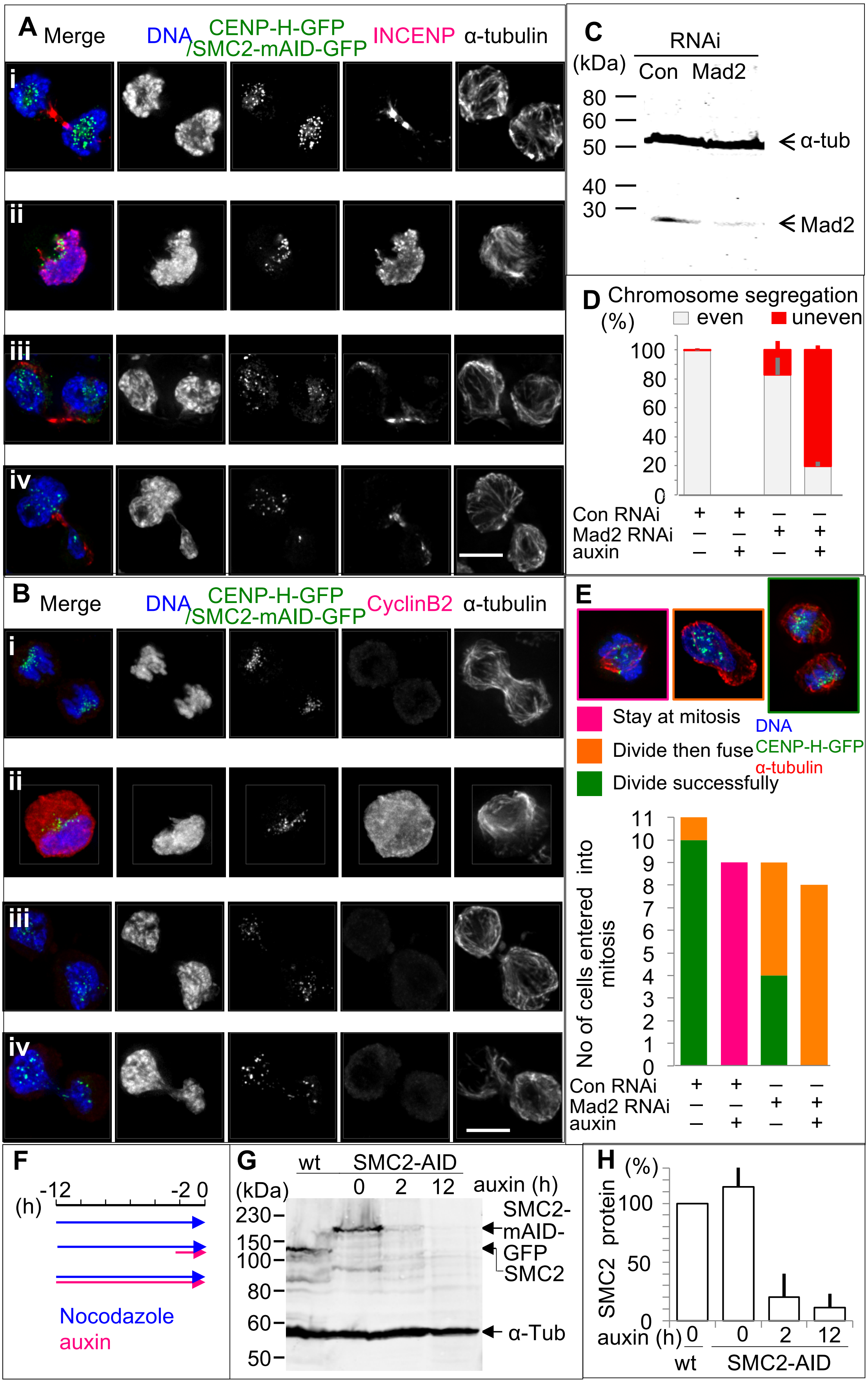

Journal of Cell Science • Supplementary information

Fig. S4. SMC2-depleted cells exit mitosis with highly uneven chromosome segregation after Mad2 RNAi. (A) INCENP concentrates on central spindle in cells exiting mitosis. The α-tubulin image is not included in the merge. Panels show (i) no auxin, control siRNA; (ii) auxin, control siRNA; (iii) no auxin, Mad2 siRNA; (iv) auxin, Mad2 siRNA. Scale bar: 5 µm. **(B)** Cyclin B2 levels fall in cells exiting mitosis. Separate α-tubulin image is shown. Panels show (i) no auxin, control siRNA; (ii) auxin, control siRNA; (iii) no auxin, Mad2 siRNA; (iv) auxin, Mad2 siRNA. Scale bar: 5 µm. **(C)** Immunoblot analysis showing MAD2 depletion by specific siRNA. **(D)** Even/uneven chromosome segregation was scored for >50 anaphase or telophase cells per sample. Data plotted as mean ± s.d. (n=3). **(E)** Quantification of live cell imaging. Cells were transiently transfected with Histone H2B-RFP. **(F)** SMC2-AID cells were treated with nocodazole for 12 h. Auxin was added at the indicated time points. **(G)** Depletion of SMC2 was confirmed by immunoblot analysis. **(H)** Quantification of SMC2 relative to α-tubulin based on (G). Data plotted as mean ± s.d. (n=3).

Journal of Cell Science • Supplementary information

A

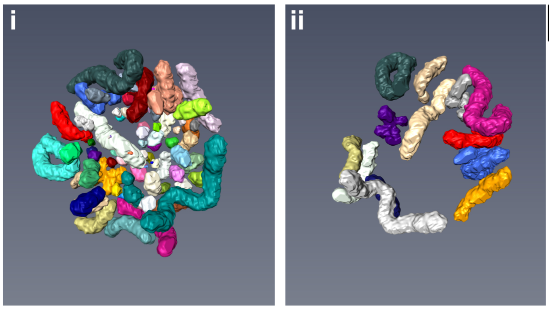

iii

Segmented Units (Chromosomes)	Predicted Chromosome	Surface Area (µm^2)	Length (µm)	Width (µm)	Volume (µm^3)	DNA content (MB)	Compaction (MB per µm^3 of unit volume)
1	1	34.22	9.05	0.94	6.43	392.41	60.99
2	1	33.62	8.91	0.91	7.36	392.41	53.32
3	2	23.80	7.4	0.97	4.91	299.12	60.93
4	2	24.03	7.25	0.89	5.02	299.12	59.58
5	3	20.20	6.15	1.02	3.82	222.60	58.31
6	3	18.03	5.78	0.94	3.78	222.60	58.93
7	4	15.44	5.61	0.84	3.01	182.57	60.64
8	4	17.71	5.45	1.14	3.65	182.57	50.00
9	5	14.99	4.07	0.96	3.08	119.65	38.85
10	5	13.04	3.81	1.02	2.66	119.65	45.02

B

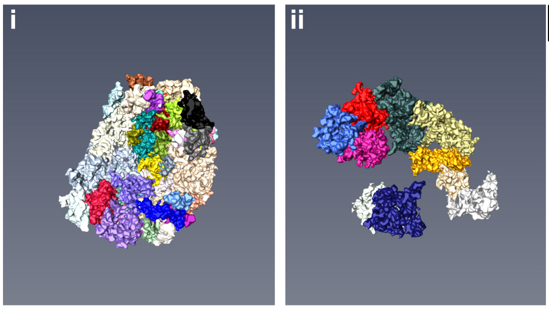

iii

Segmented Units (Chromosomes)	Predicted Chromosome	Surface Area (µm^2)	Length (µm)	Width (µm)	Volume (µm^3)	DNA content (MB)	Compaction (MB per µm^3 of unit volume)
1	?	58.17	3.98	3.78	4.70	?	?
2	?	47.19	3.89	3.89	3.86	?	?
3	?	48.77	3.77	3.48	3.61	?	?
4	?	45.70	3.66	4.01	3.56	?	?
5	?	39.12	3.48	3.46	2.82	?	?
6	?	36.46	3.45	3.23	2.70	?	?
7	?	34.02	3.31	2.96	2.62	?	?
8	?	28.82	3.16	3.15	2.61	?	?
9	?	28.73	3.01	3.2	2.16	?	?
10	?	26.85	2.97	2.76	2.13	?	?

C

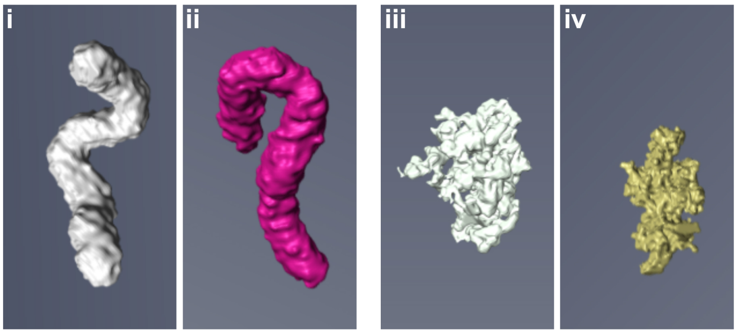

D

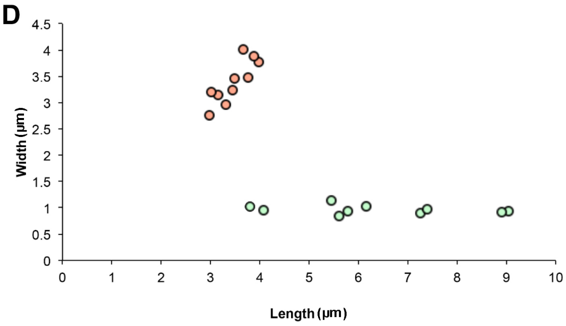

Fig. S5. Architectural parameters of chromosomes of control and SMC2 depleted chromosomes. **(A)** Control (SMC2^{on}) and **(B)** SMC2-depleted (SMC-AID auxin) cells were imaged by SBF-SEM. EM data was used to generate digital models in Amira. These images correspond to the cells shown in Fig. 5. Each chromosome unit was assigned a unique colour (i). The 10 largest chromosome units were identified (ii) and selected for geometric analysis (iii). **(C)** Zooms of the two largest chromosome units from control (i-ii) and SMC2-depleted (iii-iv) cells. **(D)** A 2-D plot of chromosome length vs width, for the 10 largest chromosomes identified in control (green) and SMC2-depleted (orange) cells.

Journal of Cell Science • Supplementary information

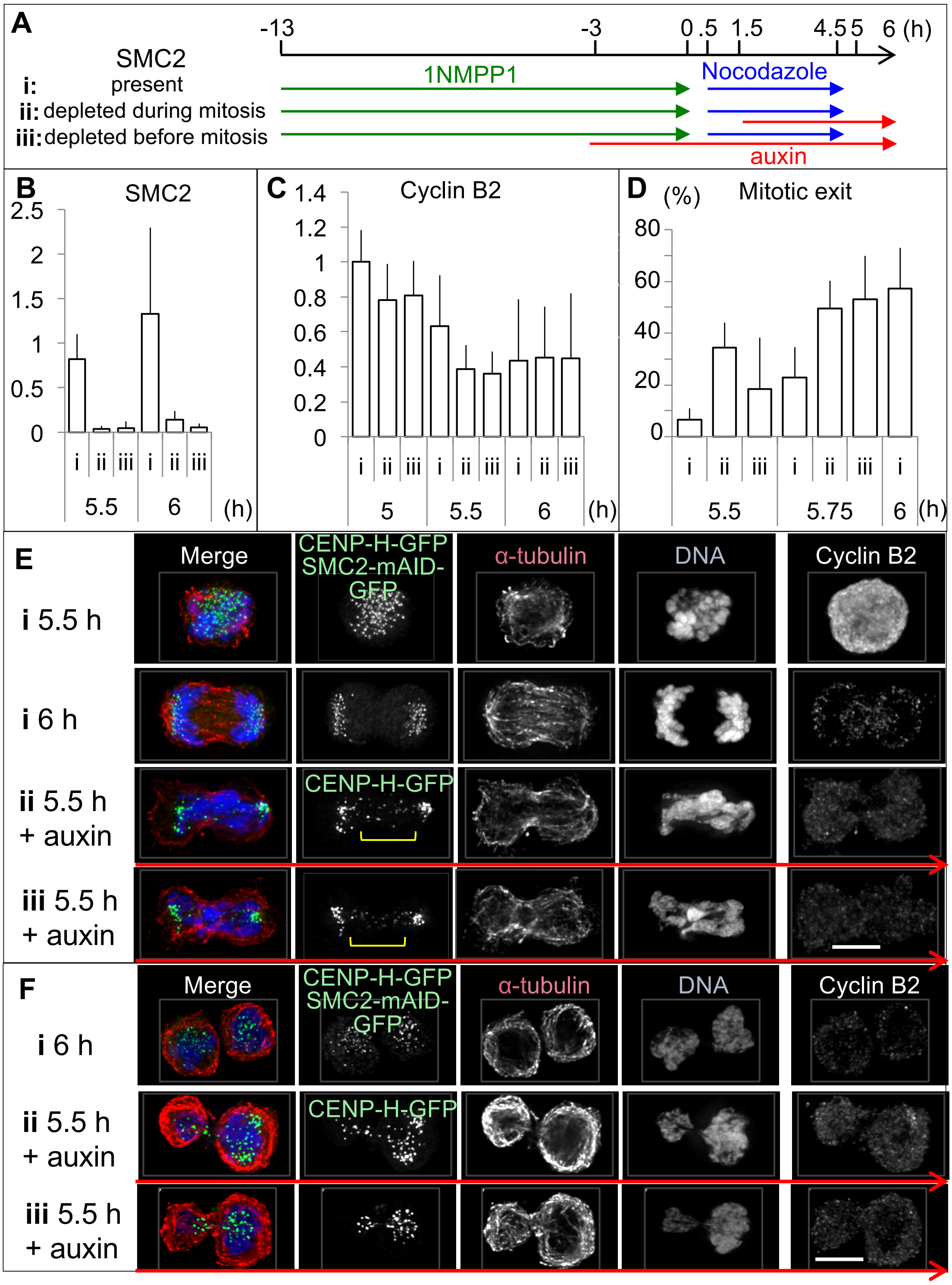

Journal of Cell Science • Supplementary information

Fig. S6. Mitotic exit of cells depleted of SMC2. (A) Experimental protocol. **(B)** The relative amount of SMC2 was measured by immunoblot analysis using anti-SMC2 and anti α-tubulin antibodies. Data plotted as mean ± s.d. (*n*=3). **(C)** The relative amount of Cyclin B2 was measured by immunoblot analysis using anti-Cyclin B2 and anti α-tubulin antibodies. Data plotted as mean ± s.d. (*n*=3). **(D)** Cells were fixed with 4 % formaldehyde at the time points indicated and stained with DAPI before counting. > 100 cells/sample were counted. Data plotted as mean ± s.d. (*n*=3). **(E)** Cells were fixed with formaldehyde in PBS without hypotonic treatment at the indicated time points and stained with anti-tubulin (red), Cyclin B2 antibodies (grey) and DAPI (blue). CENP-H-GFP + SMC2-mAID-GFP are green. The Cyclin B2 image is not included in the merge. Scale bar: 5 µm.

Supplementarymovies

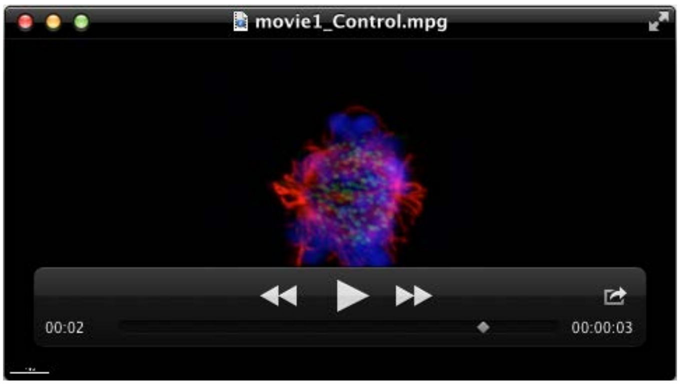

(1) 3D reconstruction of a control cell shown in Fig. 3A taken by 3D-SIM microscopy. DNA (blue), CENP-H-GFP (green), α-tubulin (red).

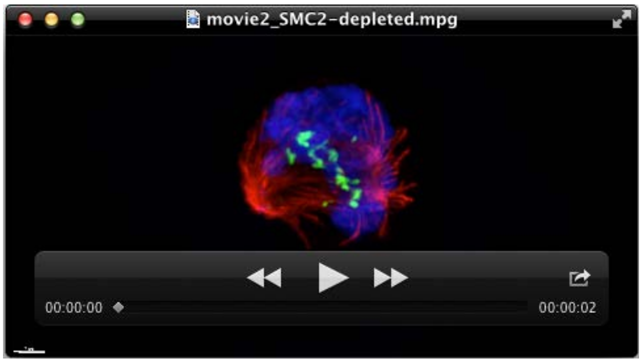

(2) 3D reconstruction of a SMC2-depleted cell shown in Fig. 3A taken by 3D-SIM microscopy. DNA (blue), CENP-H-GFP (green), α-tubulin (red).

Journal of Cell Science • Supplementary information

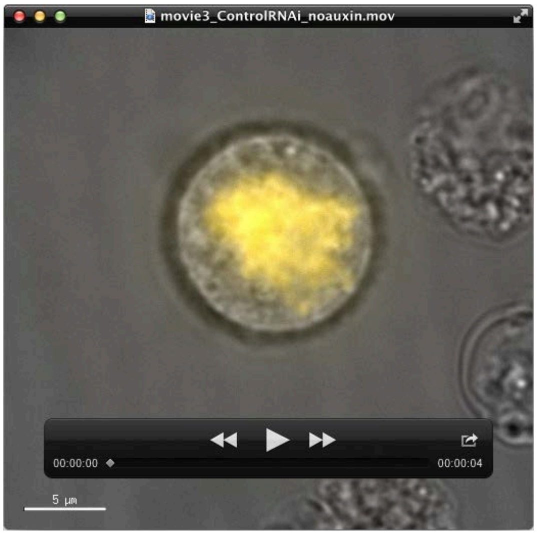

(3) SMC2-AID/CDK1^{as} cells were transiently transfected with Control siRNA oligo and an expression plasmid for H2B-RFP. After release from 4 h 1NMPP1 treatment (no auxin), those cells were imaged every 5 minutes using Deltavision microscopy. H2B-RFP (yellow) and phase contrast (grey). Scale Bar: 5 µm.

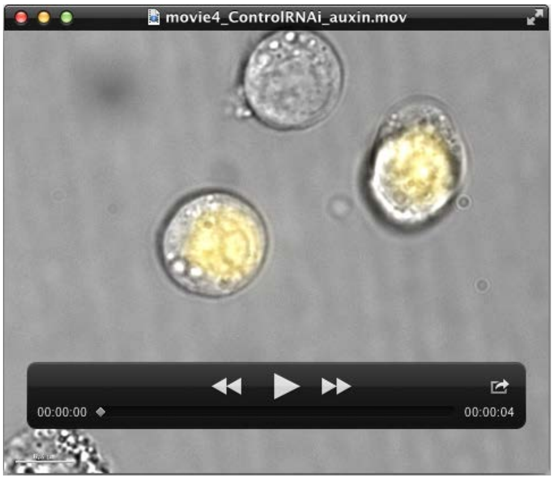

(4) SMC2-AID/CDK1^{as} cells were transiently transfected with Control siRNA oligo and an expression plasmid for H2B-RFP. After release from 4 h 1NMPP1 treatment (including 3 h auxin treatment), those cells were imaged every 5 minutes using Deltavision microscopy. H2B-RFP (yellow) and phase contrast (grey). Scale Bar: 5 µm.

Journal of Cell Science • Supplementary information

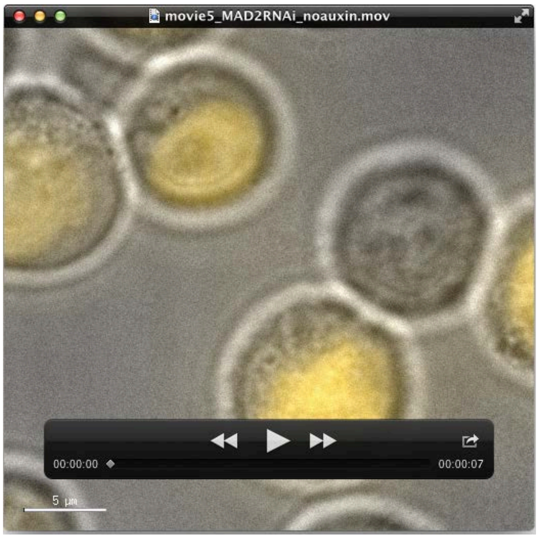

(5) SMC2-AID/CDK1^{as} cells were transiently transfected with Mad2 siRNA oligo and an expression plasmid for H2B-RFP. After release from 4 h 1NMPP1 treatment (no auxin), those cells were imaged every 5 minutes using Deltavision microscopy. H2B-RFP (yellow) and phase contrast (grey). Scale Bar: 5 µm.

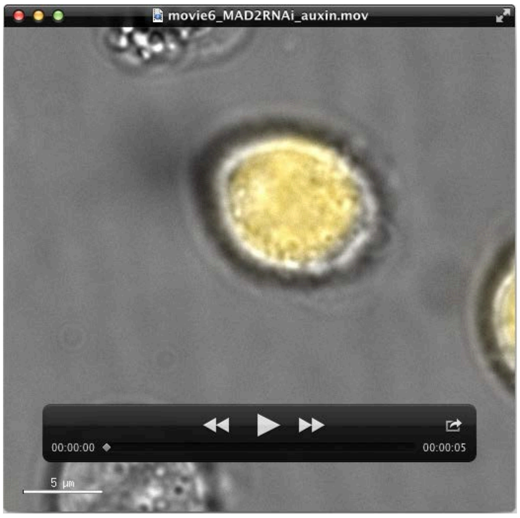

(6) SMC2-AID/CDK1^{as} cells were transiently transfected with Mad2 siRNA oligo and an expression plasmid for H2B-RFP. After release from 4 h 1NMPP1 treatment (including 3 h auxin treatment), those cells were imaged every 5 minutes using Deltavision microscopy. H2B-RFP (yellow) and phase contrast (grey). Scale Bar: 5 µm.

Journal of Cell Science • Supplementary information
